# Homologous Sequential Immunization Using Salmonella Oral Administration Followed by an Intranasal Boost with Ferritin-Based Nanoparticles Enhanced the Humoral Immune Response against H1N1 Influenza Virus

**DOI:** 10.1128/spectrum.00102-23

**Published:** 2023-05-08

**Authors:** Zhannan Wang, Tongyu Zhang, Futing Jia, Chongbo Ge, Yingkai He, Yawen Tian, Wenfeng Wang, Guilian Yang, Haibin Huang, Jianzhong Wang, Chunwei Shi, Wentao Yang, Xin Cao, Yan Zeng, Nan Wang, Aidong Qian, Chunfeng Wang, Yanlong Jiang

**Affiliations:** a College of Animal Medicine, Jilin Provincial Engineering Research Center of Animal Probiotics, Jilin Provincial Key Laboratory of Animal Microecology and Healthy Breeding, Engineering Research Center of Microecological Vaccines (Drugs) for Major Animal Diseases, Ministry of Education, Jilin Agricultural University, Changchun, China; Changchun Veterinary Research Institute

**Keywords:** influenza virus, nanoparticle vaccine, *Salmonella*, *in situ*, sequential immunization, T_RM_ cell

## Abstract

The influenza virus continues to pose a great threat to public health due to the frequent variations in RNA viruses. Vaccines targeting conserved epitopes, such as the extracellular domain of the transmembrane protein M2 (M2e), a nucleoprotein, and the stem region of hemagglutinin proteins, have been developed, but more efficient strategies, such as nanoparticle-based vaccines, are still urgently needed. However, the labor-intensive *in vitro* purification of nanoparticles is still necessary, which could hinder the application of nanoparticles in the veterinary field in the future. To overcome this limitation, we used regulated lysis Salmonella as an oral vector with which to deliver three copies of M2e (3M2e-H1N1)-ferritin nanoparticles *in situ* and evaluated the immune response. Then, sequential immunization using Salmonella-delivered nanoparticles followed by an intranasal boost with purified nanoparticles was performed to further improve the efficiency. Compared with 3M2e monomer administration, Salmonella-delivered *in situ* nanoparticles significantly increased the cellular immune response. Additionally, the results of sequential immunization showed that the intranasal boost with purified nanoparticles dramatically stimulated the activation of lung CD11b dendritic cells (DCs) and elevated the levels of effector memory T (T_EM_) cells in both spleen and lung tissues as well as those of CD4 and CD8 tissue-resident memory T (T_RM_) cells in the lungs. The increased production of mucosal IgG and IgA antibody titers was also observed, resulting in further improvements to protection against a virus challenge, compared with the pure oral immunization group. Salmonella-delivered *in situ* nanoparticles efficiently increased the cellular immune response, compared with the monomer, and sequential immunization further improved the systemic immune response, as shown by the activation of DCs, the production of T_EM_ cells and T_RM_ cells, and the mucosal immune response, thereby providing us with a novel strategy by which to apply nanoparticle-based vaccines in the future.

**IMPORTANCE** Salmonella-delivered *in situ* nanoparticle platforms may provide novel nanoparticle vaccines for oral administration, which would be beneficial for veterinary applications. The combination of administering Salmonella-vectored, self-assembled nanoparticles and an intranasal boost with purified nanoparticles significantly increased the production of effector memory T cells and lung resident memory T cells, thereby providing partial protection against an influenza virus challenge. This novel strategy could open a novel avenue for the application of nanoparticle vaccines for veterinary purposes.

## INTRODUCTION

The influenza virus still poses great threats to public health. Considering the significant antigen drift among different influenza virus strains, the need to develop a universal vaccine against the influenza virus has drawn increasing attention in recent decades (1). Such universal influenza vaccines would significantly reduce global morbidity and mortality from seasonal influenza epidemics while also protecting populations against the potential emergence of novel pandemic influenza viruses from animal reservoirs, including swine and poultry (2).

Nanoparticles ranging in size between 1 and 100 nm or up to 1,000 nm have recently been considered to be an attractive option for combatting infectious diseases (3). In vaccinology, nanoparticles serve three major roles as adjuvants, carriers, or presentation platforms, depending on the interaction between the antigen and the nanoparticle (4). The use of nanoparticles for vaccines has been extensively reported, and the advantage is attributable to the nanoscale particle size, which facilitates uptake by antigen-presenting cells, thereby leading to efficient antigen recognition and presentation (5). There are different nanovaccines, such as virus-like particles (VLPs) (6), proteinaceous nanoparticles (self-assembled protein nanocages and bacteriophages) (7), and synthetic nanoparticles (liposomes) (8). Among these, ferritin is a major iron storage protein in the body, and it consists of 24 subunits that self-assemble to form spherical nanocages that are approximately 12 nm in diameter. Ferritin, with its multimeric nature, ease of genetic modification, easy handling, and large-scale production, certainly has potential in vaccine development (9). In particular, Helicobacter pylori (H. pylori) ferritin has been commonly used in vaccine design due to its sequence divergence from human ferritin (3), and a number of antigens have been fused to the N terminus of ferritin, such as the HA protein of influenza virus (10), the spike protein receptor-binding domain of SARS-CoV2 (11), and eODGT8 from HIV (10).

Considering the severe antigenic drift of the influenza virus, the desire for a conserved influenza vaccine targeting the well-known conserved antigens HA2 (12), nucleoprotein (13), M2e (14), and M1 (15) have been extensively reported. Furthermore, nanovaccines targeting conserved influenza virus antigens have also been recently described. Ximena Zottig et al. described the self-assembly of a chimeric peptide comprised of a 10-mer β-sheet sequence and a highly conserved epitope that was derived from the influenza A virus (M2e) and produced 100 to 200 nm long uniform nanorods (NRs) that displayed the M2e epitope on their surfaces. Upon intranasal immunization in combination with the polymeric adjuvant Montanide gel, the M2e-NRs conferred complete protection against a lethal experimental infection with the H1N1 influenza A virus, displaying an absence of clinical signs (14). A similar study also showed that three sequential repeats of M2e (3M2e) could be presented on the self-assembling recombinant human heavy chain ferritin (rHF) cage to yield 3M2e-rHF nanoparticles. Intranasal vaccination with 3M2e-rHF nanoparticles induced robust immune responses, including high titers of serum M2e-specific IgG antibodies, T cell immune responses, and mucosal secretory IgA antibodies in mice, which provided efficient protection against both H1N1 and H9N2 lethal virus challenges (16).

However, although the designed nanovaccines have been reported to induce efficient protection against homosubtypic and heterosubtypic influenza virus challenges, they are still mainly dependent on the *in vitro* production and purification of synthesized nanoparticles. Recently, a novel *in situ* DNA-launched nanovaccine platform, namely, DLnano (10), was reported to display an HIV immunogen that spontaneously self-assembles *in vivo* to yield the *in vivo* production of nanovaccines. The DLnano vaccines induce stronger humoral responses than do their monomeric counterparts in both mice and guinea pigs, and they uniquely elicit CD8^+^ effector T cell immunity, compared to recombinant protein nanovaccines (10). Similar results were also reported in a cancer-related study. Electroporation-facilitated DLnano-vaccines that scaffold immunodominant melanoma Gp100 and Trp2 epitopes were shown to induce more potent and consistent epitope-specific cytolytic T cell responses than were the corresponding DNA monomeric vaccines or CpG-adjuvanted peptide vaccines (17). Notably, this novel technology still relies on the *in vitro* purification of plasmid DNA instead of a protein as well as on a special electronic instrument called the CELLECTRA 3P device (Inovio Pharmaceuticals) for the efficient transfection of plasmid DNA *in vivo* (10), which could possibly hinder the use of this technology in veterinary applications.

A number of bacterial vectors, such as attenuated Salmonella (18) and probiotic lactic acid bacteria (19), have been used to deliver protective antigens through an oral immunization approach. In particular, Roy Curtiss III’s lab developed a series of Salmonella strains and vectors with novel features to enhance immunogenicity, including regulated delayed synthesis of heterologous antigens (20) and regulated delayed attenuation (21), which provide the vaccine strain with a nearly wild-type ability to colonize lymphoid tissues before exhibiting an attenuated phenotype, thereby leading to the development of strong immune responses. Another unique strategy is regulated delayed programmed lysis, which is designed to facilitate antigen release via cell lysis within the immunized animal and to confer biological containment (22). After oral immunization in mouse and chicken models, Salmonella could replicate for 6 to 10 cycles *in vivo* and then gradually produce antigens and lyse in host cells due to the absence of arabinose, thereby resulting in the release of the synthesized antigen (22–24) or DNA vaccine (25, 26). At this moment, the idea of generating an *in situ* nanoparticle vaccine using regulated delayed lysis Salmonella as a delivery vehicle has drawn our attention. In fact, a recent study (27) showed that attenuated Salmonella could be used to deliver the capsid protein (CAP) of porcine circovirus type 2 (PCV2). The synthesized CAP protein self-assembled into VLPs in the recombinant attenuated S. Choleraesuis and induced a CAP-specific Th1-dominant immune response, mucosal immune responses, and neutralizing antibodies against PCV2. However, it is still not clear whether Salmonella can be used to deliver a self-assembled protein (ferritin)-based nanoparticle vaccine for veterinary applications. If such a strategy is successful, this will provide us with an elegant platform by which to produce cost-effective and low-cost nanovaccines by simply fusing the desired antigen with self-assembled proteins for oral immunization, without the necessity to purify nanoparticles *in vitro*.

Recently, sequential immunization has drawn increasing attention, especially due to the SARS-CoV-2 pandemic. Primer immunization with an inactivated SARS-CoV-2 vaccine that is followed by a booster vaccination using a subunit vaccine or an adenoviral vectored vaccine usually resulted in a dramatically increased systemic immune response and in better protection in a number of animal models and clinal trials against SARS-CoV-2 challenges (28, 29). Inspired by the exciting results of sequential immunization, we were also interested in determining whether a similar prime-boost strategy could be employed with our newly designed *in situ* nanoparticle vaccines against influenza virus.

In this study, we used a ferritin-based, self-assembled nanovaccine technology in combination with regulated delayed synthesis and a lysis Salmonella vector to determine whether the *in situ* production of 24-mer nanoparticles could improve immunogenicity, compared with the 3M2e monomer antigen. In addition, a sequential immunization approach applying oral administration with an intranasal boost was also performed to evaluate its immune-enhancing effects, compared with traditional, pure oral immunization.

## RESULTS

### The regulated expression of 3M2e-ferritin and the characterization of 3M2e-ferritin nanoparticles.

**FIG 1:**
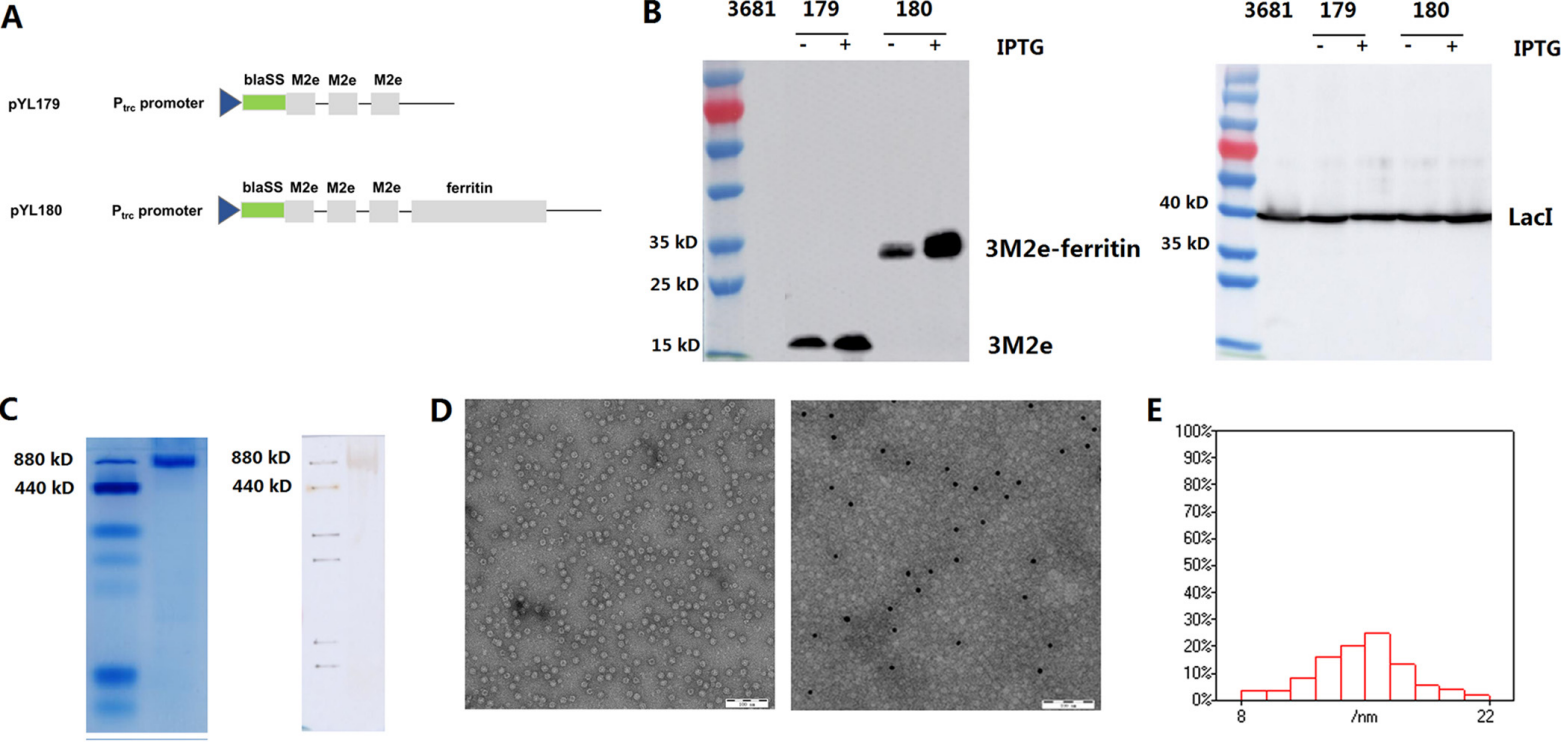
Plasmid construction, protein synthesis, and characteristics of the desired nanoparticles. Three copies of M2e were inserted into the parental plasmid pYA3681, either alone or fused with ferritin, and this yielded the plasmids pYL179 and pYL180 (A). The LacI-regulated P_trc_ promoter and type 2 secretion signal sequence of β-lactamase (bla-SS) were designed to facilitate antigen secretion. The expression of the M2e and LacI proteins in Salmonella strains was determined by Western blotting with (+) or without (−) 1 mM IPTG (B). The formation of 24-mer nanoparticles was then determined by pseudonative PAGE, nonreducing Western blotting (C), and transmission electron microscopy (TEM) analyses (D_left_). The localization of M2e outside the nanoparticles was then confirmed by immune electron microscopy (D_right_). The average size of the nanoparticles was further determined by a TEM analysis of at least 300 individual particles (E).

Three copies of M2e(H1N1) were linked together using the (GGGGS)_3_ linker, and they were inserted into the parental plasmid pYA3681, either alone or fused together with the N terminus of H. pylori ferritin, to yield pYL179 and pYL180 (Fig. 1A), respectively. To facilitate the secretion of the synthesized antigen, an optimized type 2 secretion signal sequence of β-lactamase (bla-SS) (23) was designed to be located between the LacI-regulated P_trc_ promoter and the desired antigens. An attenuated Salmonella strain (χ11802) with regulated delayed expression and lysis characteristics (23) was used in this study for oral immunization. To achieve regulated delayed expression, LacI was controlled under the arabinose-regulated *araC* P_BAD_ promoter in the Salmonella chromosome, which could inhibit the activity of the P_trc_ promoter during plasmid construction. In the presence of arabinose in the culture medium, the production of the LacI protein (approximately 38 kDa) was confirmed in all strains (Fig. 1B, right panel), with almost similar amounts to those shown in the Western blotting results. To further confirm the regulated delayed expression, the cell pellets from both cultures, with or without IPTG induction, were subjected to Western blotting. The protein bands equal to 3M2e (approximately 16 kDa) and 3M2e-ferritin (approximately 35 kDa) were detected as shown in the left panel of Fig. 1B. Notably, the presence of IPTG could repress the inhibition between LacI and the P_trc_ promoter, thereby resulting in the increased production of the desired proteins under the P_trc_ promoter, compared with that observed in the absence of IPTG. However, it is worth mentioning that there was still some leaky expression, even without IPTG, indicating that the *araC* P_BAD_-LacI regulator was not as strict as expected.

To confirm the production of the self-assembled 3M2e-ferritin nanoparticles in Salmonella, the purified proteins were analyzed, as described in Materials and Methods, by nonreducing SDS–PAGE, Western blotting, transmission electron microscopy (TEM), and immune electron microscopy. Nonreducing SDS-PAGE and Western blotting using an anti-M2 monoclonal antibody were performed, and the results showed that a protein band at approximately 840 kDa was present, and it was equal to the size of the 24-mer ferritin nanoparticle (Fig. 1C), indicating that the presence of 3M2e did not affect the self-assembly of ferritin into 24-mer nanoparticles. The presence of ferritin nanocages was also confirmed by TEM (Fig. 1D, left panel). In addition, an immune electron microscopy analysis using an anti-M2 monoclonal antibody was performed, and the results showed the presence of gold-labeled black spots on the surfaced of the nanoparticles (Fig. 1D, right panel), which confirmed the presence of M2e peptides on the outside. The average sizes of the nanoparticles were further determined to be 15.0 ± 1.4 nm via a TEM analysis that included at least 300 individual particles (Fig. 1E).

### 3M2e-ferritin nanoparticles induced the increased production of intracellular cytokines, as shown by a flow cytometry analysis.

**FIG 2:**
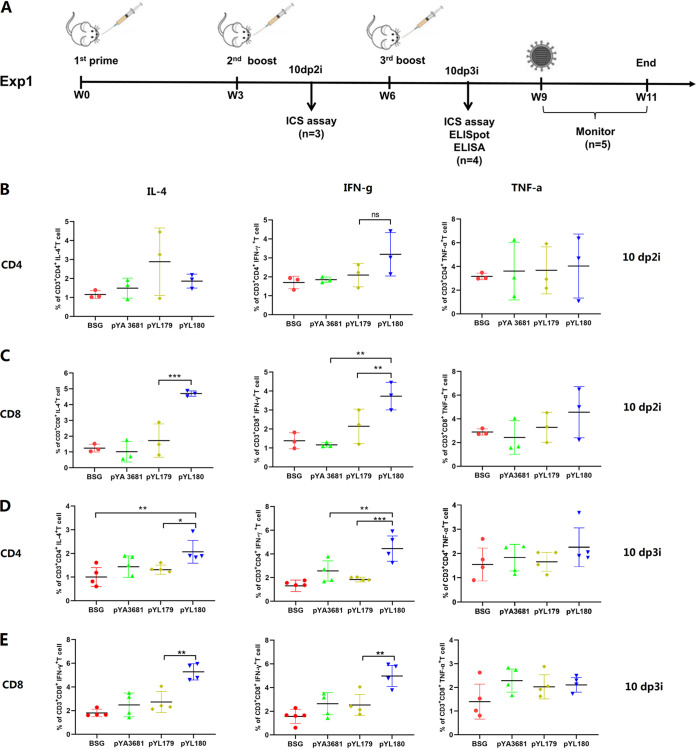
Animal experimental design (Experiment 1) and intracellular production of IL-4, IFN-γ, and TNF-α in CD4 and CD8 T cells. An illustration of the animal experiment (Experiment 1) is shown (A). C57BL/6 mice were orally administered the appropriate material three times at three-week intervals. The intracellular cytokine (ICS) assay was performed at 10 days post-second immunization (10 dp2i) and 10 days post-third immunization (10 dp3i) by fluorescence-activated cell sorting (FACS). An ELISpot and an ELISA were also conducted at 10 dp3i. The remaining mice were challenged with 0.5 LD_50_ H1N1 virus 9 weeks after the first immunization, and their body weights were monitored for 2 weeks. The single-cell suspensions from the spleen samples were subjected to FACS to determine the intracellular cytokine (IL-4, IFN-γ, and TNF-a) production in both the CD4^+^ cells (B) and the CD8^+^ (C) T cells at 10 dp2i (*n* = 3) and 10 dp3i (D and E) (*n* = 4; *, *P *< 0.05; **, *P *< 0.01; ***, *P *< 0.001).

Previous studies have demonstrated that the *in vivo* production of nanoparticles by an electroporation-mediated DNA launched platform could elicit dramatically increased levels of the CD8^+^ T cell-mediated cellular immune response, compared with either *in vivo*-produced soluble monomer antigen components (17) or *in vitro*-produced protein nanoparticles (10). To clarify whether a similar enhancement in the cellular immune response could be elicited by Salmonella-delivered *in situ* nanoparticles, a mouse study named Experiment 1 was performed using χ11802 strains harboring pYL179 and pYL180 for oral immunization as indicated (Fig. 2A). The production of intracellular cytokines from spleens was evaluated via an intracellular cytokine staining (ICS) assay (Fig. S1) to determine the production of cytokines (IL-4, IFN-γ, and TNF-α) from both CD4 and CD8 T cells upon stimulation with the synthesized M2e peptide at 10 days post-second immunization (10 dp2i) (Fig. 2B and C) and 10 days post-third immunization (10 dp3i) (Fig. 2D and E). The results showed that the generation of 3M2e-ferritin nanoparticles in the pYL180 group significantly increased the production of IFN-γ in CD8^+^ T cells, compared with pYL179 at 10 days post-second immunization (10 dp2i) (*P *< 0.01) (Fig. 2C) or 10 days post-third immunization (10 dp3i) (*P *< 0.01) (Fig. 2E), indicating an enhanced Th1-type immune response. Similar results were also observed in that the production of nanoparticles seemed to increase the production of CD8^+^ IL-4^+^ T cells at both 10 dp2i (*P *< 0.001) (Fig. 2C) and 10 dp3i (*P *< 0.01) (Fig. 2E). Regarding the generation of intracellular IL-4 in CD4 T cells, an increased proportion of CD4^+^ IL-4^+^ T cells in pYL180-immunized mice at 10 dp3i, compared with pYL179-immunized mice, was also observed (*P *< 0.05) (Fig. 2D), although there was no significant difference between these two groups at 10 dp2i (Fig. 2B), indicating that there was also a trend for the 24-mer nanoparticle to stimulate the Th2 subtype response. On the other hand, the production of TNF-α in both CD4 (Fig. 2B and D) and CD8 (Fig. 2C and E) T cells did not show any significant difference throughout the whole experiment.

### The increased production of IFN-γ^+^ lymphocytes, as shown by an ELISpot assay.

**FIG 3:**
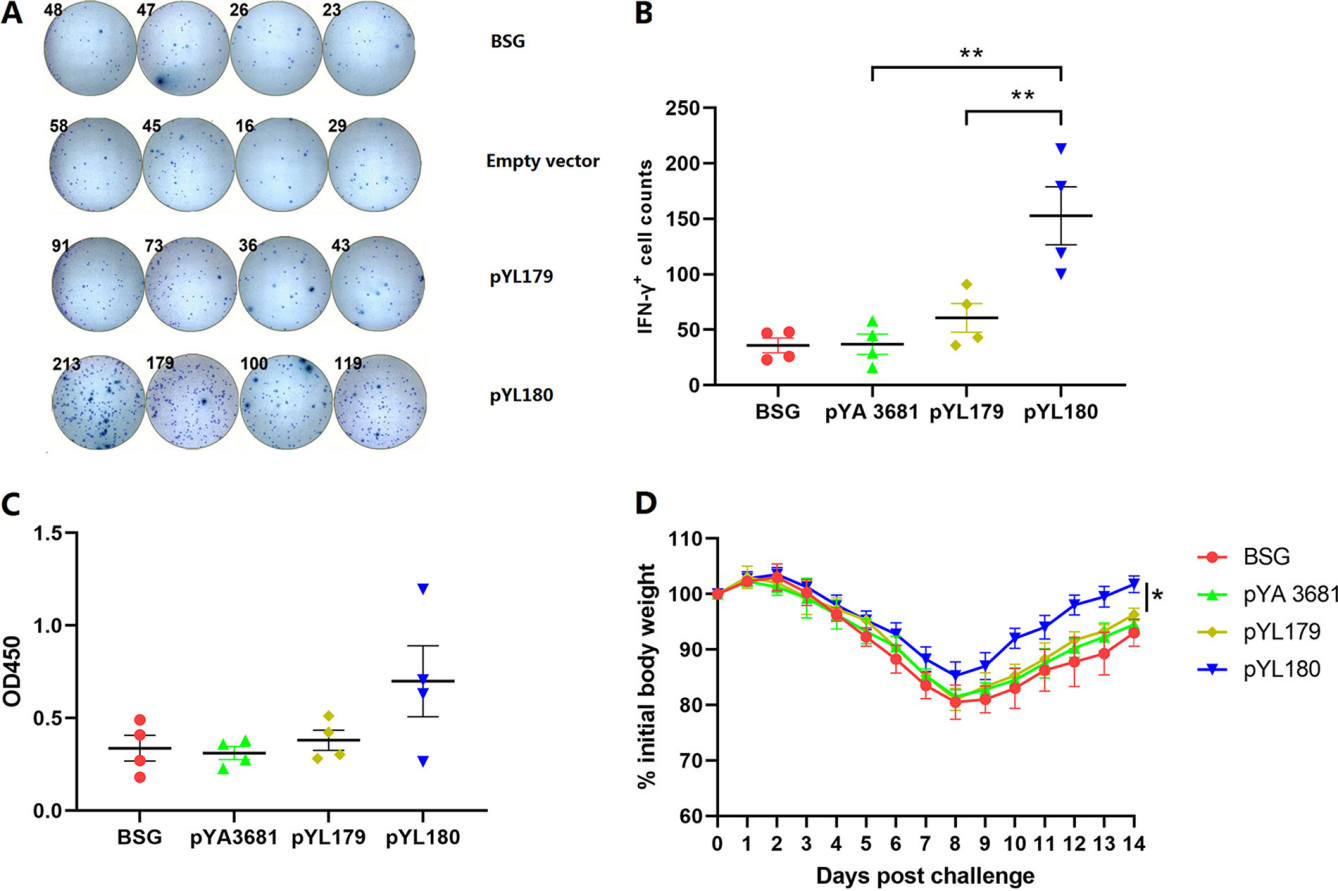
ELISpot assays were used to determine the IFN-γ-positive lymphocytes and H1N1 influenza challenge study. The single-cell suspensions from the spleen samples were subjected to ELISpot assays to determine the numbers of IFN-γ positive lymphocytes at 10 days post-third immunization (10 dp3i) (A and B) (*n* = 4; **, *P* < 0.01). Serum samples were also collected at 10 dp3i, and the M2e-specific IgG antibody titers were measured via ELISA (*n* = 4) (C). The remaining mice were then challenged with 0.5 LD_50_ H1N1 virus, and their body weight gains were recorded for 14 days (*n* = 5) (D).

An ELISpot assay was performed to further confirm the increased cellular immune response that was caused by the *in situ* production of 24-mer nanoparticles. Whereas the IFN-γ-positive spots were not observed to be any different among the buffered saline with gelatin (BSG), empty vector, and pYL179 monomer groups, which was consistent with the previous intracellular cytokine production that was shown via fluorescence-activated cell sorting (FACS) (Fig. 2), there were obviously increased numbers of IFN-γ-positive spots in the spleen samples of pYL180 24-mer immunized mice (Fig. 3A) after incubation with the synthesized H1N1-specific M2e peptide. A statistical analysis showed that the presence of 24-mer nanoparticles in the pYL180 group significantly increased the number of IFN-γ-positive spots, compared with either the monomer pYL179 (*P *< 0.01) or empty vector pYA3681 (*P *< 0.01) (Fig. 3B), thereby confirming the increased cellular immune response induced by the nanoparticles.

### A modest serum IgG antibody response was induced by the 24-mer nanoparticles.

Serum samples were collected and analyzed via ELISA to determine the M2e-specific IgG antibody response. Oral immunization with attenuated Salmonella harboring the 24-mer nanoparticle-producing plasmid pYL180 did not elicit remarkable serum IgG antibody titers, compared with the 3M2e monomer plasmid pYL179, even though there was a trend for increased antibody production in pYL180-immunized mice, and no significant difference was identified (Fig. 3C). These data were somewhat consistent with the results of previous reports that indicated that M2e was a relatively poor immunogen with which to elicit a humoral immune response (30, 31). A low-dose challenge study was then performed, and the results showed that no dramatic protection was observed in 3M2e-ferritin nanoparticle-immunized mice, compared with 3M2e monomer-immunized mice, although the mice recovered sooner on day 8 post-challenge (Fig. 3D), indicating that Salmonella-delivered *in situ* 3M2e-ferritin nanoparticles alone may not be adequate to elicit the expected protection.

### An intranasal boost with purified nanoparticles induced recruitment and the differentiation of CD11c^+^ CD11b^+^ DCs in the lungs.

**FIG 4:**
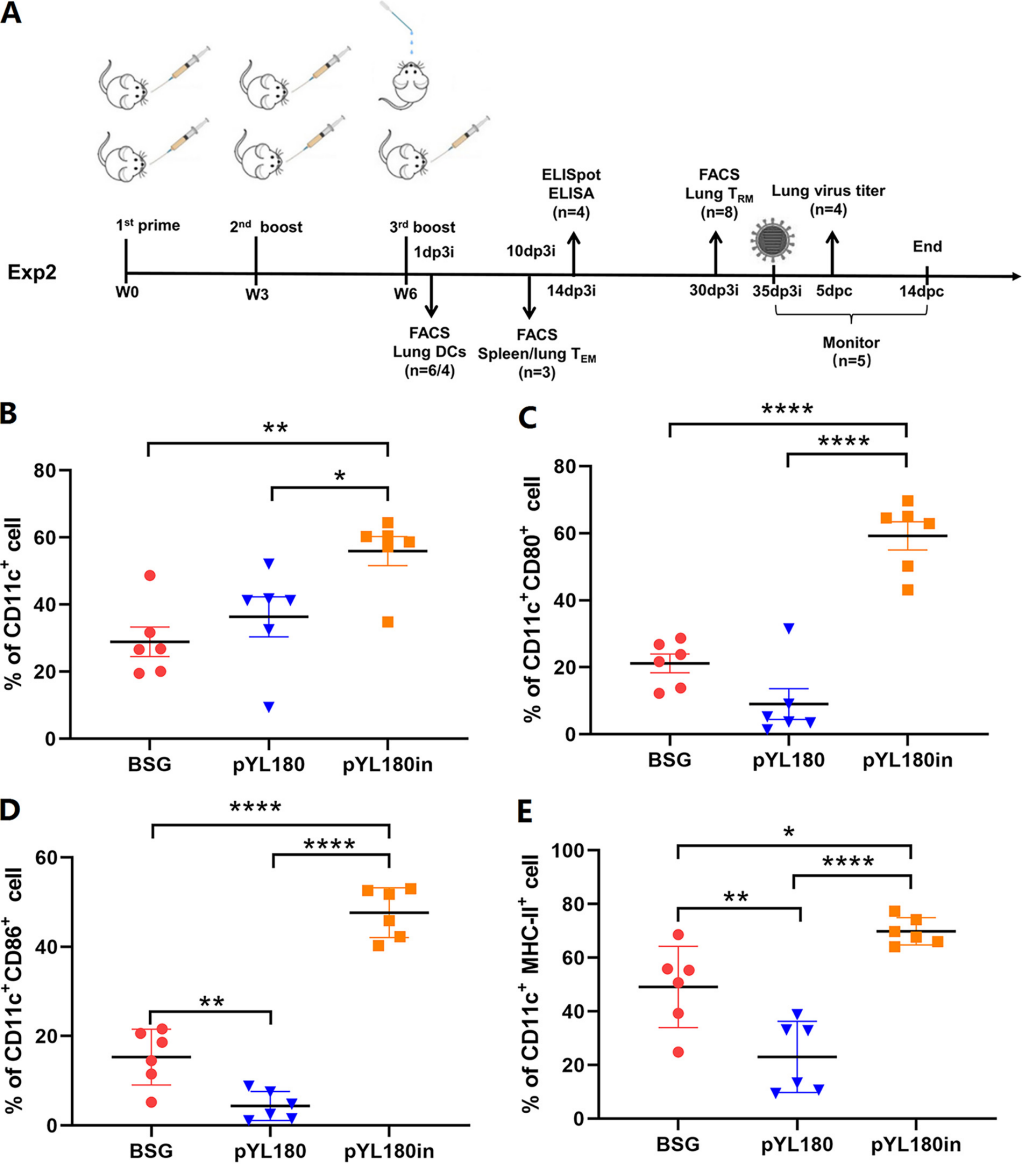
The animal experimental design (Experiment 2) and the differentiation of CD11c^+^ DCs in the lungs of mice. An illustration of the animal experiment (Experiment 2) is shown (A). dp3i, days post-third immunization; FACS, fluorescence-activated cell sorting; dpc, days post-challenge. Single-cell suspensions from the lung samples were collected and subjected to FACS to determine the percentage of CD11c^+^ DCs (B) and the differentiation markers, namely, CD80 (C), CD86 (D), and MHC-II (E) at 1 dp3i (*n* = 6; *, *P *< 0.05; **, *P *< 0.01; ***, *P *< 0.001).

Since we were not satisfied with the lower serum antibody titers, a sequential boost immunization using the purified nanoparticles (Experiment 2) was performed to determine whether this procedure could stimulate a more effective humoral immune response. Mice were treated with either three doses of Salmonella or two doses of Salmonella with one additional intranasal boost with purified protein, as shown in Fig. 4A. The lung lymphocytes were analyzed via FACS at 1 day post-third immunization (1 dp3i) to determine whether intranasal immunization could affect DCs in mouse lung tissues (Fig. S2). The results showed that intranasal immunization resulted in the significantly increased recruitment of CD11c^+^ DCs, compared with the three oral immunization group (pYL180) (*P *< 0.05) or the BSG control group (*P *< 0.01) (Fig. 4B). Similar trends were also observed regarding the differentiation markers, which demonstrated that intranasal immunization could significantly increase the percentage of CD11c^+^ CD80^+^ (Fig. 4C), CD11c^+^ CD86^+^ (Fig. 4D), and CD11c^+^ MHC-II^+^ (Fig. 4E) DCs. Notably, we also observed interesting results, as differentiation significantly decreased in the pYL180 oral immunization group, compared with the BSG control group, especially in terms of CD86 (*P *< 0.01) (Fig. 4D) and MHC-II (*P *< 0.01) (Fig. 4E) levels.

**FIG 5:**
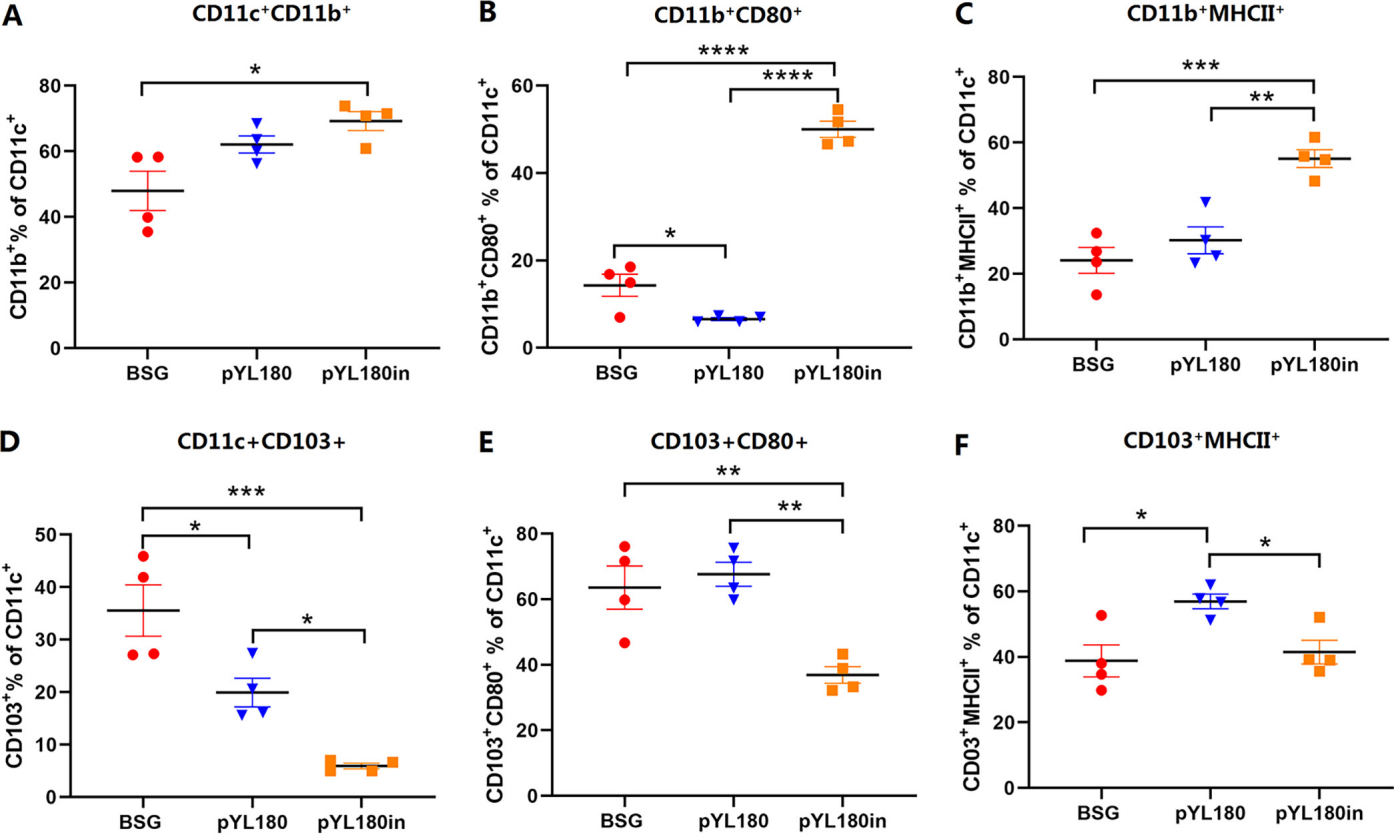
Populations and differentiation of CD11c^+^ CD11b^+^ DCs and CD11c^+^ CD103^+^ DCs in lung tissue. Single-cell suspensions from lung samples were subjected to FACS to determine the percentage of CD11c^+^ CD11b^+^ DCs (A). The differentiation of CD11b^+^ DCs was also determined by detecting the surface markers, namely, CD80 (B) and MHC-II (C). Moreover, the percentages of CD11c^+^ CD103^+^ (D), CD11c^+^ CD103^+^ CD80^+^ (E), and CD11c^+^ CD103^+^ MHCII^+^ (F) DCs were determined (*n* = 4; *, *P *< 0.05; **, *P *< 0.01; ***, *P *< 0.001; ****, *P *< 0.0001).

To further determine which subtype of the DCs was responsible for the observed reaction of the lung DCs, the percentages of conventional CD11c^+^ CD103^+^ DCs (cDC1) and CD11c^+^ CD11b^+^ DCs (cDC2), as well as their differentiations, were evaluated via FACS (Fig. S3). The results demonstrated a significantly increased percentage of cDC2 in the pYL180in (*P *< 0.05) groups, compared with the BSG control (Fig. 5A). Regarding the differentiation markers CD80 and MHC-II, the third intranasal boost resulted in a significantly increased population of CD11c^+^ CD11b^+^ CD80^+^ (Fig. 5B) and CD11c^+^ CD11b^+^ MHC-II^+^ (Fig. 5C) cells, compared with both the BSG and pYL180 groups, indicating that intranasal immunization resulted in the increased development of CD11b DCs. On the other hand, the percentages of cDC1 were dramatically decreased in the pYL180in group, compared with the BSG (*P *< 0.001) and pYL180 (*P *< 0.05) groups (Fig. 5D). Interestingly, the percentages of CD103^+^ CD80^+^ DCs (*P *< 0.01) (Fig. 5E) and CD103^+^ MHCII^+^ DCs (*P *< 0.05) were also significantly decreased, compared with those in the pYL180 groups (Fig. 5F). All of these observations indicated that the intranasal boost using 24-mer nanoparticles mainly resulted in the recruitment of cDC2 from the circulatory system to the lung, and this was followed by increased differentiation and maturation, which could be responsible for the further enlargement of the humoral immune response.

### Intranasal immunization elicited the expansion of CD44^+^ CD62L^−^ effector memory CD4^+^ T cells in both the spleen and the lung.

**FIG 6:**
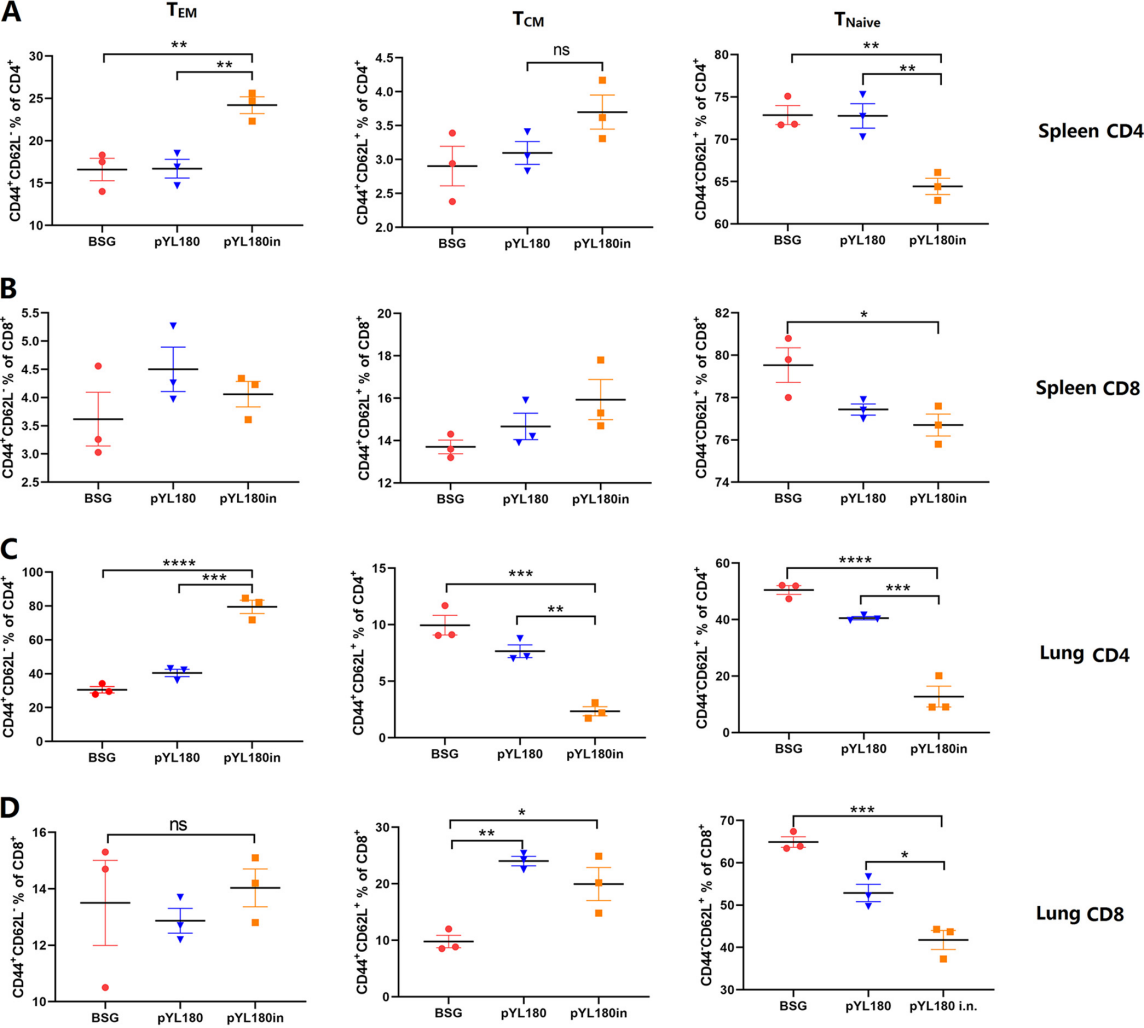
Generation of T_EM_, T_CM_, and T_Naive_ effector/memory T cells in spleen and lung tissues. Single-cell suspensions from the spleen (A and B) and lung tissues (C and D) were subjected to FACS to determine the percentages of CD44^high^ CD62L^low^ T_EM_ cells, CD44^high^ CD62L^high^ T_CM_ cells, and CD44^low^ CD62L^high^ T_Naive_ cells at 10 days post-third immunization in Experiment 2 (*n* = 3; *, *P *< 0.05; **, *P *< 0.01; ***, *P *< 0.001; ****, *P *< 0.0001; ns, not significant).

The surface markers CD44 and CD62L (l-selectin) were used to define three major subsets of T cells in mice: naive (CD44^−^ CD62L^+^ [T_Naive_]), central memory (CD44^+^ CD62L^+^, [T_CM_]), and effector/memory (CD44^−^ CD62L^+^, [T_EM_]) (32). Lymphocytes were collected on day 10 post-third immunization and were analyzed via FACS to determine the percentages of T_EM_, T_CM_, and T_Naive_ cells in the spleens (Fig. S4) and lungs (Fig. S5). The results showed that the percentages of CD4^+^ CD44^+^ CD62L^−^ T_EM_ cells in the spleens of pYL180-immunized mice were significantly increased, compared with those in the spleens of both BSG-immunized and pYL180-immunized mice (*P *< 0.01) (Fig. 6A). Moreover, the percentages of CD4^+^ CD44^−^ CD62L^+^ T_Naive_ cells in the pYL180in group were significantly decreased, compared with those in the other two groups (*P *< 0.01). The increased formation of CD4^+^ CD44^+^ CD62L^+^ T_CM_ cells was also observed in the spleens of the pYL180in group, although this was without a significant difference (Fig. 6A). Regarding CD8^+^ T_EM_ and T_CM_ cells in spleen samples, no significant differences were observed among all three groups; however, there was a significantly decreased percentage of T_Naive_ cells in the pYL180in group, compared with that observed with the BSG control (*P *< 0.05) (Fig. 6B).

Similar trends were also observed regarding the frequencies of T_EM_ and T_Naive_ cells in the lung samples. The intranasal boost with purified 3M2e-ferritin nanoparticles significantly increased the production of CD4^+^ CD44^+^ CD62L^−^ T_EM_ cells and decreased the percentages of CD4^+^ CD44^−^ CD62L^+^ T_Naive_ cells in lung lymphocytes (Fig. 6C), and this was consistent with the spleen sample observations (Fig. 6A). However, a significantly decreased percentage of CD4^+^ CD44^+^ CD62L^+^ T_CM_ cells was also noticed in the pYL180in group, which was different from the previous spleen sample observations (Fig. 6A). The results for the CD8^+^ T cell subset were similar to those from the spleen, which demonstrated that the percentage of CD8^+^ T_EM_ cells was not significantly different among all three groups, even though decreased T_Naive_ and increased T_CM_ cell production could be distinguished (Fig. 6D), similar to that observed with the spleen samples (Fig. 6B).

### Intranasal immunization resulted in the significantly increased production of lung-resident memory T (T_RM_) cells.

**FIG 7:**
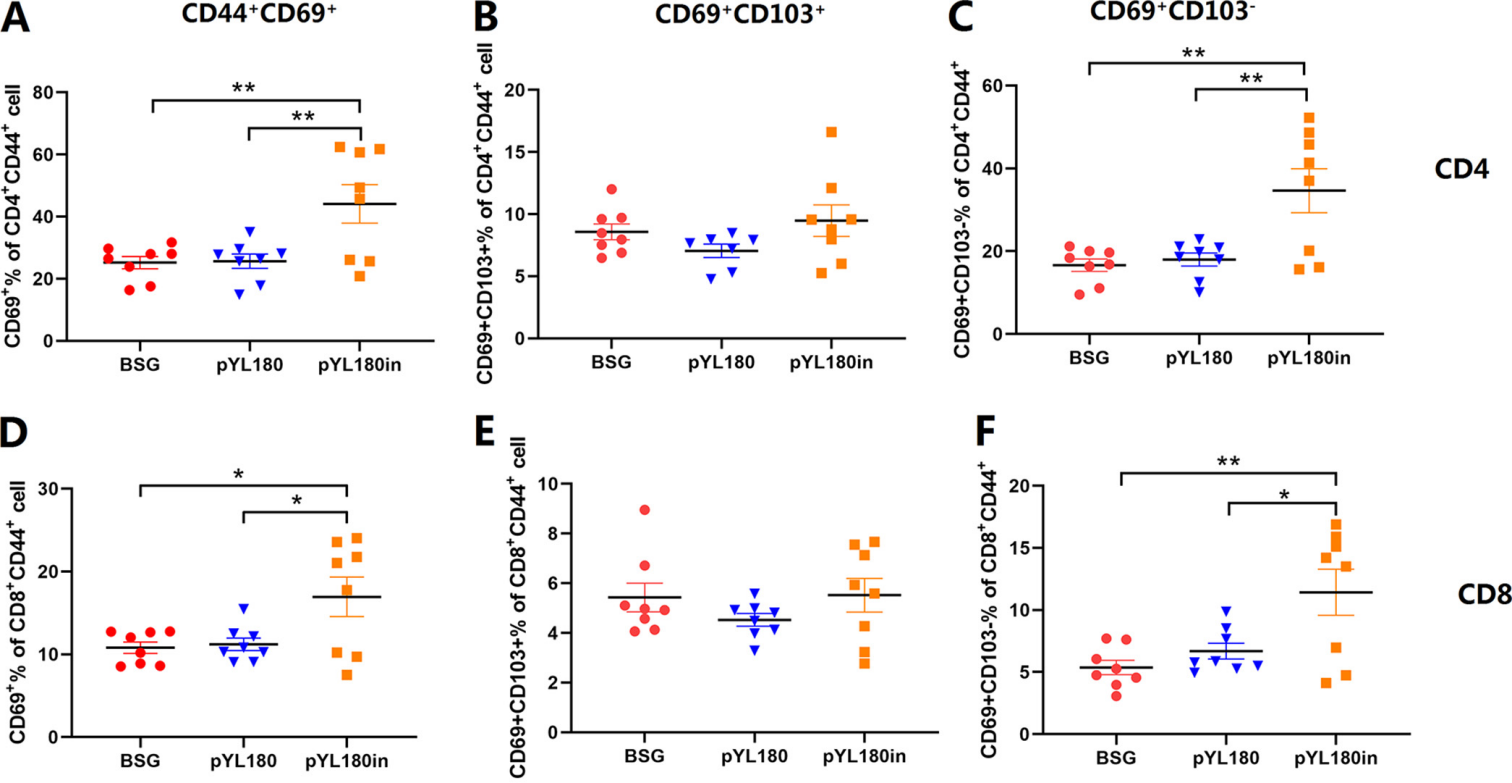
Formation of CD4^+^ CD44^+^ CD69^+^ and CD8^+^ CD44^+^ CD69^+^ lung-resident memory T (T_RM_) cells in lung tissues. Single-cell suspensions from the lung samples were subjected to FACS to determine the percentages of CD4^+^ CD44^+^ CD69^+^ (A), CD4^+^ CD44^+^ CD69^+^ CD103^+^ (B), CD4^+^ CD44^+^ CD69^+^ CD103^−^ (C), CD8^+^ CD44^+^ CD69^+^ (D), CD4^+^ CD44^+^ CD69^+^ CD103^+^ (E), and CD8^+^ CD44^+^ CD69^+^ CD103^−^ (F) T_RM_ cells at 30 days post-third immunization in Experiment 2, using specific antibodies (*n* = 8; *, *P *< 0.05; **, *P *< 0.01).

Naive T cells can differentiate into effector T cells by antigenic stimulation. Some effector cells become memory cells, which quickly give rise to effector cells in response to the same antigenic stimulation (32). It has been demonstrated that intranasal immunization could induce lung T_RM_ cells, which could be critical for protection against a virus challenge (33–35). To determine whether the intranasal boost using purified 24-mer 3M2e-ferritin nanoparticles could elicit the production of lung T_RM_ cells, single-cell suspensions of lung tissue were subjected to FACS (Fig. S6), and the results showed that the intranasal boost (pYL180in) significantly increased the production of CD4^+^ CD44^+^ CD69^+^ T_RM_ cells, compared with that observed in both the BSG and pYL180 groups (*P *< 0.01) (Fig. 7A). To be more specific, the percentages of CD103^+^/CD103^−^ T cells were also measured, and the results demonstrated that the CD69^+^ CD103^+^ T cells were not obviously affected in any of the three groups (Fig. 7B), whereas the number of CD4^+^ CD44^+^ CD69^+^ CD103^−^ T cells significantly increased in the pYL180in groups, compared with the BSG and pYL180 groups (*P *< 0.01) (Fig. 7C).

Similar trends were also observed regarding the CD8^+^ T_RM_ population. The intranasal boost with nanoparticles significantly increased the production of CD8^+^ CD44^+^ CD69^+^ T_RM_ cells, compared with that observed in both the BSG and pYL180 groups (*P *< 0.05) (Fig. 7D). Again, no obvious differences were observed among all three groups for the CD69^+^ CD103^+^ T cells (Fig. 7E). On the other hand, the populations of CD8^+^ CD44^+^ CD69^+^ CD103^−^ T cells were significantly increased, compared with those in the BSG (*P *< 0.01) and pYL180 groups (*P *< 0.05) (Fig. 7F).

### The intranasal boost with 3M2e nanoparticles increased the levels of mucosal antibodies and intracellular IFN-γ, compared with traditional oral administration.

**FIG 8:**
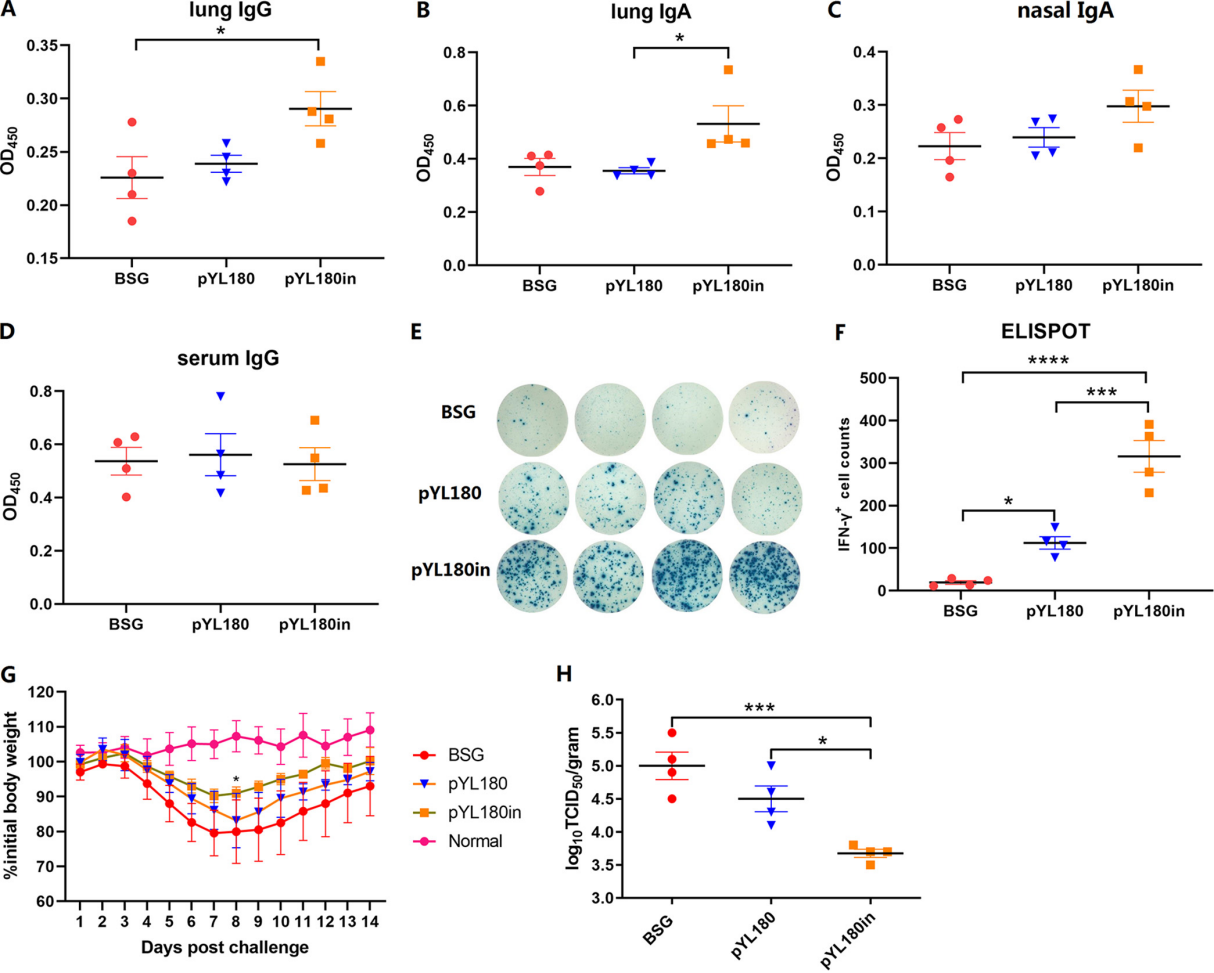
Production of M2e-specific antibodies and the intracellular cytokine IFN-γ. ELISAs were performed to measure the M2e-specific lung IgG (A), lung IgA (B), nasal IgA (C), and serum IgG (D) antibodies at 14 days post-third immunization (14 dp3i) (*n* = 4). An ELISpot was also conducted to determine the intracellular IFN-γ levels (E and F). Then, the remaining mice (*n* = 9 per group) were challenged at 35 days post-third immunization (35 dp3i), and their body weight gains were recorded for 2 weeks (G). Lung samples were collected from 4 mice per group at 5 days post-challenge (dpc), and the virus titers were determined via a TCID_50_ analysis using MDCK cells (H) (*, *P *< 0.05; ***, *P *< 0.001).

Serum IgG as well as mucosal IgG and IgA antibody production was evaluated. The results showed that the intranasal boost with 3M2e nanoparticles appeared to increase the antibody titers regarding lung IgG (Fig. 8A), lung IgA (Fig. 8B), and nasal IgA (Fig. 8C), especially regarding the stimulation of the production of lung IgA, compared with the pYL180 oral immunization group (*P *< 0.05) (Fig. 8B). However, the serum-specific M2e antibody titers were not significantly different among all three groups (Fig. 8D), indicating that the intranasal boost had a greater effect on the mucosal immune response than on the humoral immune response in C57BL/6 mice. ELISpot assays were also performed to determine the production of intracellular IFN-γ (Fig. 8E). The results demonstrated that the intranasal boost significantly increased the frequency of IFN-γ-producing cells in the spleen, compared with both the pYL180 oral immunization group (*P *< 0.001) and the BSG control group (*P *< 0.0001), whereas in the pure pYL180 oral immunization group, the level of cellular IFN-γ also appeared to significantly increase, compared with that observed in the BSG group (*P *< 0.05) (Fig. 8F). After a sublethal H1N1 challenge, the body weights in the pYL180in group decreased slower than did those in both the BSG and pYL180 groups, reaching their lowest values at day 7 postinfection, whereas the body weights in both the BSG and pYL180 groups continued to decrease until day 8 before beginning to recover (Fig. 8G). Significant differences were observed between the pYL180 and pYL180in groups on day 8 postchallenge (Fig. 8G). The virus titers in the lung samples on day 5 postchallenge were also determined via TCID_50_ assays, and the results showed that the virus titers in the pYL180in groups were significantly decreased, compared with those in both the BSG control (*P *< 0.001) and the pYL180 groups (*P *< 0.05) (Fig. 8H).

## DISCUSSION

Antigen-focused nanoparticle vaccines have been considered to be more efficient in eliciting antigen-specific immune responses, especially CD8 T cell-mediated cellular immune responses, than monomer soluble antigens. Notably, electroporation-facilitated, DNA-launched *in situ* nanoparticle vaccination (DLnano-vaccines) has been demonstrated to induce stronger neutralizing antibodies (36) and robust CD8^+^ T cell immunity (10, 17), whereas the bolus deliveries of protein nanovaccines that were followed by electroporation failed to induce CTLs, suggesting that the direct *in vivo* production of nanovaccines may be critical in the stimulation of cellular immunity. In particular, both *in vivo* production and the formation of nanoparticles could be necessary to elicit an effective cellular response, as the DLnano-vaccines that were scaffolding immunodominant melanoma Gp100 and Trp2 epitopes were shown to induce more potent and consistent epitope-specific CTL responses than did the corresponding monomeric DNA vaccines (17).

In this study, we used the regulated delayed lysis Salmonella strain χ11802 as a vehicle with which to deliver an *in situ* nanoparticle vaccine. The lysis strain was designed to include the regulated delayed synthesis of the desired antigen as well as regulated delayed lysis after immunization due to the absence of arabinose *in vivo* (23), thereby resulting in increased efficiency in inducing a specific humoral immune response in both chicken (24, 37) and mouse (26, 38) studies, as found previously. The synthesized 3M2e-ferritin protein could self-assemble into a 24-mer nanocage and then be released into the host environment during Salmonella lysis to yield the desired *in situ* nanoparticles, as expected. Without the need for *in vitro* purification and electroporation-based immunization, the Salmonella-delivered *in situ* nanoparticle platform would be more appropriate for veterinary applications, considering economic and realistic factors. Similar to the previously described DLnano-vaccines (10, 17), the Salmonella vectored *in vivo* nanoparticles also elicited significantly increased cellular immune responses, as shown by the intracellular cytokine production by both FACS and ELISpot assays. CD8^+^ IFN-γ^+^ T cells and CD8^+^ IL-4^+^ T cells have been identified as Tc1 and Tc2 cytolytic effector cells, respectively (39, 40). Whereas Tc1 cells have been considered to be the predominantly cytolytic cells during the cellular immune response, Tc2 cells appear to have a controversial role in the immune response. Some reports have indicated that Tc2 cells show only a low level of cytotoxicity, compared with Tc1 cells (41), whereas other studies have suggested that Tc2 cells are highly cytotoxic during influenza infection and autoimmune diabetes (42). Moreover, the adoptive transfer of either Tc1 or Tc2 CD8^+^ effector T cell subpopulations can effectively induce tumor cell regression and can subsequently prolong survival times in mice bearing established pulmonary malignancies (39, 43). As shown in Fig. 2, the *in situ* production of 3M2e-ferritin nanoparticles induced both Tc1 and Tc2 cells upon stimulation with the M2e peptide, thereby indicating the possible stimulation of both subtypes of Tc cells being induced by the Salmonella-mediated *in situ* nanovaccine. However, although pYL180 immunization stimulated higher titers of serum IgG antibodies than did pYL179 immunization, no significant differences were identified (Fig. 3C). One of the possible explanations for this result could be that M2e is usually considered to be a poor immunogen (30, 31).

Sequential immunization has drawn increasing attention recently, especially in the field of COVID-19 vaccination. Heterologous COVID-19 vaccination has been demonstrated to generate higher antibody and cell-mediated immune responses than have homologous vaccination regimens (44, 45). Two doses of inactivated vaccine (KCONVAC, China, Shenzhen Kangtai) that were followed by a heterologous booster with Ad5-nCoV elicited a strikingly higher level of neutralizing antibodies against the Delta (B.1.617.2) and Omicron (B.1.1.529) variants, a dramatic upregulation of CD86 on cDC1, an increased level of polyfunctional T cells, and an elevated T follicular helper cell response (29). To determine whether the immunity of Salmonella-delivered *in situ* nanovaccines could be improved by sequential immunization, a homologous intranasal boost with purified 3M2e nanoparticles was administered. In general, the intranasal boost increased the levels of the specific mucosal IgA and IgG antibodies in both nasal washing and lung washing samples, especially that of the lung IgA. However, the serum IgG antibody titer was still not dramatically affected in the C57BL/6 mouse model (Fig. 8). In fact, a similar oral prime and intranasal boost experiment was performed in our laboratory using BALB/c model mice, and the results demonstrated that the intranasal boost significantly increased the antigen-specific serum IgG and vaginal IgA antibody titers, compared with those in the oral immunization group (Fig. S7), indicating that the mouse species possibly affects the humoral immune response. In fact, different immune responses between C57BL/6 and BALB/c mice have been reported previously, regarding the M2e-specific humoral immune response using a DNA prime-recombinant adenovirus (rAd) boost vaccination approach (46) in which the results demonstrated that BALB/c mice developed a strong immune response against M2e, whereas C57BL/6 mice did not, due to both MHC differences (H-2d versus H-2b) and background genes. Another possible reason could be due to the properties of C57BL/6 mice, which are appropriate for the induction of a Th1-dominant cellular immune response instead of a Th2-biased humoral immune response (47). Therefore, to improve the humoral immune response that was stimulated by Salmonella-mediated *in situ* nanoparticles, other strategies, such as the presence of an effective adjuvant, more efficient antigens, such as hemagglutinin (48), and a dendritic cell targeting strategy (49), could be involved.

The transformation from T_EM_ cells to T_RM_ cells has been described previously. Upon a respiratory pathogen encounter, DCs migrate to the mediastinal lymph node to activate naive CD8^+^ T cells, by which effector CD8^+^ T cells then migrate into the nasal or lung tissue for their conversion into T_RM_ cells (50). In the lungs, local tissue factors, including antigens, cytokines, costimulation, and cellular interactions, together with a tissue-resident transcriptional profile, drive the formation of T_RM_ cells, whereas the maintenance of parenchyma lung CD8^+^ T_RM_ cells has been proposed to be dependent on replenishment from circulating CD8^+^ T_EM_ cells (50). In this study, we observed that CD11c^+^ DCs, especially CD11c^+^CD11b^+^ DCs in the lungs, could be efficiently activated by intranasal boost immunization (Fig. 4 and 5). Then, the activated and mature DCs migrate from the lung to the draining mediastinal lymph node, where the T cells interact with mature DCs bearing specific antigens and become activated. Newly activated T cells proliferate and begin to acquire effector functions, including the ability to produce effector cytokines that direct other immune functions, thereby resulting in the generation of increased percentages of T_EM_ cells. In contrast to T_EM_ and T_CM_ cells, which circulate throughout the peripheral and secondary lymphoid tissues, respectively, recent studies have highlighted the tissue-specific nature of the T_RM_ subset (51). T_EM_ cells have the highest conversion rate to T_RM_ cells *in vitro* and *in vivo* (52). Considering the dramatically increased formation of T_EM_ cells in the spleen and lungs at 10 dp3i (Fig. 6) and of T_RM_ cells in the lungs at 30 dp3i in pYL180in-immunized mice, there could be a possible conversion from T_EM_ to T_RM_ cells during the transfer of T_EM_ cells from the spleen to the lungs, through which they encounter the foreign 3M2e nanoparticles after intranasal immunization (Fig. 7). Notably, different numbers of mice have been selected in this study for some specific considerations. For example, during Experiment 2, different numbers were noticed between the experiments of CD11c DCs (*n* = 6) and CD11b DCs (*n* = 4) as well as between the experiments that were used to determine T_EM_ (*n* = 3) and T_RM_ (*n* = 8). Usually, we prefer at least four mice per group for more consistent results; however, sometimes three mice were used since we have repeatedly employed this type of assay in the past. Sometimes, six or eight mice were used, as we intended to make sure that the results would be acceptable and exclude possible variation due to an unfamiliarity with the operation during the experiments. For sure, more consistent animal numbers should be included for better explanations of results in our future studies.

In a recent study, Tianyang Mao et al. (53) described the development of a vaccine strategy, termed prime and spike, that leveraged existing immunity generated by primary vaccination (prime) to elicit mucosal immune memory within the respiratory tract using unadjuvanted intranasal spike boosters (spike), including robust resident memory B cell and T cell responses as well as IgA at the respiratory mucosa, which conferred complete protection against a lethal SARS-CoV-2 infection. A similar study revealed that combining intramuscular immunizations with an intranasal boost of a self-amplifying mRNA vaccine encoding the influenza A virus nucleoprotein achieved high levels of both circulating T cell memory cells and lung T_RM_ cells (54). Similar results were observed in our oral prime and intranasal boost strategy, as the intranasal boost with purified 3M2e nanoparticles significantly increased the presence of both CD4^+^ and CD8^+^ T_RM_ cells (Fig. 7). The roles of CD4^+^ and CD8^+^ T_RM_ cells in the protection against viral challenges remains controversial. Some studies have indicated that CD4^+^ T_RM_ cells can provide strong protection against a lethal challenge infection with a heterosubtypic influenza virus strain (34) or play a dominant role in the initiation of antitumor immunity (55). On the other hand, the critical role of CD8^+^ T_RM_ cells in protecting against an influenza virus infection was confirmed (56). Another study indicated that the intratracheal transfer of lung CD4^+^ and CD8^+^ T_RM_ cells conferred comprehensive protection against pneumonic plague in naive recipient mice (57). Notably, although the increased generation of both CD4^+^ and CD8^+^ T_RM_ cells in lung sections and the enhanced production of intracellular IFN-γ^+^ and mucosal IgA antibody responses were observed, the protection against a virus challenge was not satisfactory in our study. It has been demonstrated that M2e-mediated protective immunity primarily relies on nonneutralizing antibodies (58) that bind to infected cells and engage Fcγ-mediated effector mechanisms, such as antibody-dependent cell cytotoxicity (ADCC) or antibody-dependent cell phagocytosis (59, 60). Considering the relatively lower humoral IgG antibody titers that were observed with the C57BL/6 mice in our study, it may be critical to explore other strategies by which to stimulate a more efficient antibody response against the M2e peptide, such as a DCs targeting strategy (49).

In conclusion, a Salmonella-mediated *in situ* nanoparticle vaccine platform was developed, and sequential immunization using purified 3M2e-ferritin nanoparticles further improved the stimulated immune response. However, further efforts are still needed to achieve our final goal, namely, that a bacteria-delivered *in situ* nanoparticle vaccine could be efficient enough to combat severe infectious diseases.

## MATERIALS AND METHODS

### The bacterial strains, the virus, and the culture conditions.

The bacterial strains, plasmids, and virus used in this study are listed in Table 1. Escherichia coli (E. coli) χ6212 (Δasd) was used for the molecular cloning of host cells, and Salmonella strain χ11802 with a regulated delayed lysis phenotype (23) was used for immunization. Luria-Bertani medium (LB) was used for bacterial culture at 37°C, and 50 μg/mL diaminopimelic acid (DAP) (Sigma), 0.1% arabinose (Sigma), and 0.2% mannose (Sigma) were provided when necessary. The H1N1 virus (A/Puerto Rico/8/1934) (61) was used in this study. The virus was cultivated in the allantoic cavity of 9 to 11-day-old, specific pathogen-free eggs that were provided by the Harbin Veterinary Research Institute (Harbin, China). The titer of the virus was determined in C57BL/6 mice and expressed as the 50% lethal dose (LD_50_) via the Reed and Muench method (62) before use in the challenge experiment.

**TABLE 1:** Bacterial strains, plasmids, and virus used in this study

Plasmid or Strain	Description	Source
Strains		
χ11802	Lysis Salmonella strain;ΔPmurA25::TT *araC* P_BAD_ murA ΔasdA27::TT *araC* PBAD c2 Δ(wza-wcaM)-8 Δpmi-2426ΔrelA198::araC PBAD lacI TT ΔrecF126	Roy Curtiss III, University of Florida
χ6212	*E.coli* host strain for DNA cloning; asd deletion mutant	Roy Curtiss III, University of Florida
Plasmids		
pYL168	Synthesized 3M2e-ferritin gene codon optimized for expression in E. coli	This study
pYA3681	Prokaryotic expression vector in Salmonella, arabinose dependent	Roy Curtiss III, University of Florida
pYL179	Ptrc promoter expressing 3M2e monomer with bla-SS secretion signal	This study
pYL180	Ptrc promoter expressing 3M2e-ferritin fusion protein with bla-SS secretion signal	This study
Virus		
H1N1	A/Puerto Rico/8/1934	Yanlong Cong, Jilin University

### Plasmid construction.

The primers used in this study are listed in Table 2. The genes encoding three copies of the M2e (MSLLTEVETPIRNEWGCRCNGSSD) (3M2e) of the H1N1 virus fused with ferritin were codon-optimized for expression in E. coli*/*Salmonella, synthesized by GenScript (Suzhou, China), and named pYL168. The primers 3M2e-F/3M2e-R and 3M2e-F/ferritin-R were used to amplify the fragments of either 3M2e or 3M2e-ferritin, using pYL168 as the template, digesting with KpnI/SacII, and then ligating with plasmid pYL3681 that was digested with the same enzymes, thereby yielding pYL179 and pYL180 (Fig. 1A), respectively.

**TABLE 2:** Primers used in this study^*a*^

Product	Primer	Sequence	Expected size	Origin
3M2e	3M2e-F-KpnI (Forward)	GG*GGTACC*AGATGAAAAAACAAC	492 bp	This study
	3M2e-R-SacII (Reverse)	atg*CCGCGG*TTAGCTGCCCCCACCGCCAGAGC		
3M2e-ferritin	3M2e-F-KpnI (Forward)	GG*GGTACC*AGATGAAAAAACAAC	981 bp	This study
	ferritin-R-SacII (Reverse)	atg*CCGCGG*TTAGCTTTTGCGGCTTTTGG		

a Italics letters indicated the designed restriction enzymes.

### Synthesis of desired antigens.

Salmonella strain χ11802 harboring individual plasmids was shaken overnight at 37°C in LB medium supplemented with 0.1% arabinose and 0.2% mannose. The bacteria were then inoculated into fresh medium at a dilution of 1:100 and cultured further until the OD_600_ reached 0.6. After that, each strain was separated in half and induced in the presence of 1 mM IPTG for an additional 4 h, while the rest of the culture was incubated for 4 h without IPTG. At the end of the incubation, the OD_600_ values were adjusted to the same level, 1 mL of each individual strain was collected via centrifugation, and the samples were resuspended in SDS loading buffer for further Western blot assays. The rabbit polyclonal anti-LacI IgG antibody (1:1,000) (lab stock) and mouse monoclonal anti-influenza A virus M2 protein IgG antibody (14C2) (1:1,000, Abcam, United Kingdom) were used as the primary antibodies, respectively, and they were followed by individual HRP-labeled secondary antibodies (Beyotime Biotechnology, China).

### Purification of 3M2e-ferritin nanoparticles (NPs).

The purification of 3M2e-ferritin 24-mer nanoparticles from the Salmonella strain was performed, according to a previously described approach (16), with minor modifications. In detail, Salmonella strain χ11802 harboring the plasmid pYL180 was induced by 1 mM isopropyl β-d-thiogalactoside (IPTG) (Beyotime Biotechnology, China) for 16 h at 37°C. After that, the cells were collected via centrifugation, and the pellets were resuspended in assembly buffer (20 mM Tris-HCl, 50 mM NaCl [pH 8.0]) and sonicated on ice for 40 min. The solution was then centrifuged at 10,000 × *g* for 30 min to remove bacterial debris, and the supernatant was heated at 70°C for 10 min to precipitate abundant Salmonella proteins. The 24-mer nanoparticles were then purified from the collected supernatant using Anti-Flag Affinity Gel (Beyotime Biotechnology, China). The concentration of the purified protein was then determined using a BCA Protein Assay Kit (Yamei, China).

### Characteristic properties of 3M2e-ferritin nanoparticles.

The purified 3M2e-ferritin nanoparticles were evaluated via nonreducing SDS-PAGE that was followed by Western blotting using the mouse anti-influenza A M2 antibody 14C2 (Abcam, United Kingdom). The nanoparticles were characterized using transmission electron microscopy (TEM) and immune electron microscopy (service provided by the Harbin Veterinary Research Institute, Harbin, China) to confirm the presence of 3M2e on the surfaces of the ferritin nanoparticles.

In detail, nickel-coated EM grids were absorbed on drops containing 0.5 mg/mL 3M2e-ferritin cages for 2 min. After being blocked with BSA, the grids were incubated with the mouse anti-influenza A M2 antibody 14C2 (Abcam, United Kingdom), and this was followed by incubation with 5 nm colloidal gold-conjugated goat anti-mouse IgG antibodies (Thermo Fisher, USA). After being rinsed three times with 0.01 mol/L phosphate-buffered saline with Tween 20 (PBST), the grids were stained with 2% phosphotungstic acid for 1 h. Finally, the grids were dried in air and examined via TEM. A minimum of 300 discrete particles were measured from each of at least two widely separated regions of the sample, as described previously (63).

### Mouse immunization and sample collection.

All animal procedures were approved by the Institutional Animal Care and Use Committee at Jilin Agriculture University (2022 05 25 001).

**Experiment 1.** A total of 48 six to eight-week-old, pathogen-free C57BL/6 female mice were purchased from Beijing HFK Bioscience Co., Ltd., China, and were randomly divided into 4 groups, with 12 mice in each group. The Salmonella strain χ11802 harboring the empty vector pYA3681, pYL179 (3M2e), or pYL180 (3M2e-ferritin) was used for oral immunization, and BSG buffer (64) was also included as a negative control. In detail, the mice were orally immunized with recombinant Salmonella strains at a dose of 1.0 × 10^9^ CFU/0.1 mL BSG on day 0, and this was followed by two additional booster immunizations at 3-week intervals. At 10 days post-second immunization (10 dp2i), 3 mice from each group were sacrificed via CO_2_ euthanasia, and single-cell suspensions of the spleens were collected and analyzed via flow cytometry to determine the levels of intracellular cytokines (IL-4, IFN-γ, and TNF-α) (Fig. 2A). Then, at 10 days post-third immunization (10 dp3i), 4 mice from each group were sacrificed, as mentioned above, to determine their intracellular cytokine contents. In addition, the intracellular production of IFN-γ in spleen cells was also determined via an ELISpot assay, as described below. Moreover, serum samples were collected for antibody evaluation using an enzyme-linked immunosorbent assay (ELISA) (Fig. 2A). 3 weeks after the last immunization, the remaining 5 mice in each group were challenged with a dose of 0.5 LD_50_ (approximately 40 PFU) of A/Puerto Rico/8/1934 (H1N1) virus, and their body weight gains were measured for an additional 14 days.

**Experiment 2.** The second mouse study was performed to evaluate the effects of sequential immunization by the oral prime plus intranasal boost approach, compared with traditional oral immunization (Fig. 4A). A total of 102 six to eight-week-old, pathogen-free C57BL/6 female mice were randomly divided into 3 groups (*n* = 34 per group): a BSG control group, a Salmonella χ11802(pYL180) oral immunization group (named pYL180), and a χ11802(pYL180) intranasal boost sequential immunization group (named pYL180in). In detail, the mice in the χ11802(pYL180) oral immunization group were immunized with the same doses and at the same time points as those described in Experiment 1, with a total of three immunizations at three-week intervals. On the other hand, the mice in the sequential immunization group were orally inoculated with Salmonella for the first two immunizations, and this was followed by an additional intranasal boost immunization with 10 μg of 3M2e-ferritin nanoparticles that were purified from 1.0 × 10^9^ CFU Salmonella in a volume of 20 μL.

One day after the last immunization, six mice from each group were sacrificed via CO_2_ euthanasia, lung samples were collected, and single-cell samples were prepared, according to a previously described method (65). The percentage of CD11c^+^ dendritic cells (DCs) as well as the differentiation of DCs were determined via FACS, using the MHC-II, CD80, and CD86 surface markers, as described below. To further determine which subtype of DCs in the lung sections was responsible for nanoparticle capture, 4 mice from each group were also sacrificed at the same time to analyze the percentages of CD11c^+^ CD103^+^ and CD11c^+^ CD11b^+^ DC cells as well as their differentiation by evaluating the surface markers MHC-II and CD80 via FACS.

At 10 days post-third immunization, 3 mice from each group were selected randomly, and the lymphocytes from their spleens and lungs were collected to determine the percentages of CD44^+^ CD62^−^ (T_EM_), CD44^+^ CD62^+^ (T_CM_), and CD44^−^ CD62^+^ (T_Naive_) cells. At 14 days after the last immunization, 4 mice from each group were sacrificed to collect lung washing, nasal washing, and serum samples for the determination of M2e-specific IgA and IgG antibodies. The intracellular production of IFN-γ was also evaluated using an ELISpot assay, as described below. At 30 days post-third immunization, 8 mice from each group were sacrificed, and the lymphocytes from their lung samples were collected and subjected to FACS to determine the presence of lung-resident memory T (T_RM_) cells by measuring the surface markers CD3, CD4, CD8, CD44, CD69, and CD103. At 35 days after the last immunization, the remaining 9 mice were subjected to a sublethal challenge at a dose of 0.5 LD_50_ of A/Puerto Rico/8/1934 (H1N1) virus. At day 5 postchallenge, lung tissue samples were harvested from 4 mice to determine the virus titers, using a 50% tissue culture infective dose (TCID_50_) assay with Madin-Darby canine kidney (MDCK) cells, as described previously (66). The remaining 5 mice in each group were monitored, and their body weight gains were measured daily for a total of 14 days.

### Determination of specific antibody responses via ELISA.

An ELISA was performed as previously described (67), with minor modifications. Nunc Immunoplate Maxisorb F96 plates (Multi Science, Zhejiang, China) were coated with synthesized M2e peptide (MSLLTEVETPIRNEWGCRCNGSSD) from the H1N1 influenza virus (GenScript, Nanjing, China) at a concentration of 500 ng/well in coating buffer (pH 9.6) at 4°C overnight. The plates were then washed three times with PBST and blocked with PBST-BSA for 1 h at 37°C. After three washes, 100 μL of diluted serum samples (1:100) or lung washing samples (1:20) were added to each well with triplicate repeats, and a series of twofold dilutions were made. Biotinylated anti-mouse IgG or IgA antibodies (Southern Biotechnology, Birmingham, AL) that were diluted 1:10,000 and streptavidin-HRP (Southern Biotechnology, Birmingham, AL) that was diluted 1:3,000 were used to determine the antibody titers. Then, tetramethylbenzidine (TMB) was added to develop the reaction, and the color development (absorbance) was recorded at 450 nm.

### Fluorescence-activated cell sorting (FACS).

Single-cell suspensions from the spleen and lung tissues were prepared as previously described (65), with minor modifications. In detail, the spleens were gently ground in complete RPMI 1640 (10% heat-inactivated FCS [Sigma], 50 mg/mL streptomycin, and 50 U/mL penicillin [all from Invitrogen]), and the lung tissues were digested for 30 min at 37°C with 250 to 300 U/mL Collagenase Type IV (Sigma) in complete RPMI 1640 (5% heat-inactivated FCS [Sigma], 10 mM HEPES buffer, 1 mM sodium pyruvate, 2 mM l-glutamine, 10 mM β-mercaptoethanol, 50 mg/mL streptomycin and 50 U/mL penicillin [all from Invitrogen]). This was followed by sequential straining through a 70 μm filter. Then, the cells from the spleens and lungs were resuspended in red blood cell lysis buffer (Solarbio, Beijing, China) and incubated at room temperature for 2 min. The cells were washed twice with RPMI medium and diluted to the desired concentration. Then, 40 μm nylon cell strainers and 1 to 2 × 10^6^ cells were used per antibody staining reaction. FACS was then performed, using a BD Fortessa with specific antibodies from BioLegend, except for a few from BD Biosciences, as indicated.

For the T_EM_ cells, T_CM_ cells, T_Naive_ cells, and DC staining, the cells were stained by using a Zombie NIR Fixable Viability Kit (BioLegend) to gate the live cells, which were pretreated with TruStain FcX (BioLegend) so as to block the Fc receptors and stain with antibodies. Then, the following indicated antibodies were used for the T cells: PerCP/Cyanine5.5 anti-mouse CD3ε (BD Biosciences, USA), FITC-anti-CD4 antibody, Alexa Fluor 700-anti-CD8α antibody, PE/Cyanine7-anti-mouse/human CD44, and APC-anti-mouse CD62L. The following antibodies were used for the DC surface staining: Alexa Fluor 700-anti-mouse F4/80, APC-anti-mouse CD11c, Cyanine5.5-anti-mouse I-A/I-E, PE-anti-mouse CD86, PE-CF594 anti-mouse CD80 (BD Biosciences, USA), PE/Cyanine7-anti-mouse CD11b, and PE-anti-mouse CD103.

To quantify the percentages of intracellular cytokines from antigen-specific T cells, splenocytes were stimulated with 5 μg/mL M2 peptide and 2 μg/mL anti-CD28 (BioLegend) at 37°C with CO_2_ for 1 h. The cells were then incubated using Protein Transport Inhibitor (BD Biosciences, USA) for another 5 h. DMSO was used as a negative control. PMA/ionomycin was used as a positive control. After a total of 6 h, the treatment process was the same as the protocol for the surface markers, and then the cells were incubated with the reagents from a Fixation/Permeabilization Kit (BD Biosciences, USA). The following cytokine antibodies were used: PE-anti-IL-4 antibody, APC-anti-IFN-γ antibody, and PE/Cyanine7-anti-mouse TNF-α. The experiments were performed in triplicate, and data compensation and analysis were performed using FlowJo Version 7.6.1.

To evaluate the generation of T_RM_ cells in the lung sections, mice were intravenously injected with 3 μg of APC/Cy7 anti-mouse CD45 (BioLegend) diluted in 300 μL of saline at 7 min prior to euthanasia (68). Then, lung samples were collected, and single-cell suspensions were prepared as mentioned above. The following indicated antibodies were used: PerCP/Cyanine5.5 anti-mouse CD3ε (BD Biosciences, USA), FITC-anti-CD4 antibody, Alexa Fluor 700-anti-CD8α antibody, PE/Cyanine7-anti-mouse/human CD44, PE-anti-mouse CD69, and APC-anti-mouse CD103.

### ELISpot analysis.

IFN-γ-producing lymphocytes were analyzed as instructed by the manufacturer (Mabtech, Sweden), as described previously (69). In detail, the mice were euthanized at 14 dp3i, and their spleens were removed aseptically. Splenocytes were collected in complete RPMI medium (2 mM l-glutamine) supplemented with 10% bovine serum by gently agitating the spleens between two microscope slides. The resulting suspension was passed through a 70 mm cell strainer, and the cells were washed two times with PBS. After the second wash, the cells were resuspended in red blood cell lysis buffer (Solarbio, Beijing, China) and incubated at room temperature for 2 min. The cells were washed twice with RPMI medium and diluted to the desired concentration. Twofold dilutions of 2.5 × 10^5^ cells/well were cultured in wells that had been coated with IFN-γ capture antibodies. The cells were incubated for 30 h and were either stimulated with 5 mg/mL M2e peptides or left unstimulated. After 30 h, the splenocytes were removed, and IFN-γ secretion was detected, as recommended by the manufacturer. The ELISpot plates were analyzed using an ImmunoSpot Analyzer by CTL (Cellular Technology Ltd.). The plates were air-dried, prior to analysis. The results were analyzed using an ImmunoSpot Analyzer from CTL (Cellular Technology Ltd., USA). Spot forming units (SFU), each representing the secretory footprint of a single IFN-γ secreting T cell, were automatically counted using ImmunoSpot Software (CTL), which relies on a statistics-based, automatic setting of upper and lower size gates for SFU counting.

### Statistical analysis.

All data are displayed as the mean ± the SEM (standard error of the mean). Prism 8.3 (GraphPad) was used to perform a one-way analysis of variance (ANOVA) and Tukey’s post-test for the statistical analysis. A *P* value of <0.05 was considered to be indicative of a statistically significant result.

### Data availability.

All of the data supporting our findings in this study are available either within the article and its supplemental information files or from the corresponding author upon request (yanlong.jiang@jlau.edu.cn).
