# Supplementary material for: Homologous Sequential Immunization Using Salmonella Oral Administration Followed by an Intranasal Boost with Ferritin-Based Nanoparticles Enhanced the Humoral Immune Response against H1N1 Influenza Virus
Source: Microbiol Spectr. 2023 May 8;11(3):e00102-23. doi: 10.1128/spectrum.00102-23 (PMC10269571; doi:10.1128/spectrum.00102-23)
Supplement: Supplemental file 1 — Supplemental material. Download spectrum.00102-23-s001.docx, DOCX file, 2 MB [file spectrum.00102-23-s001.docx]

**
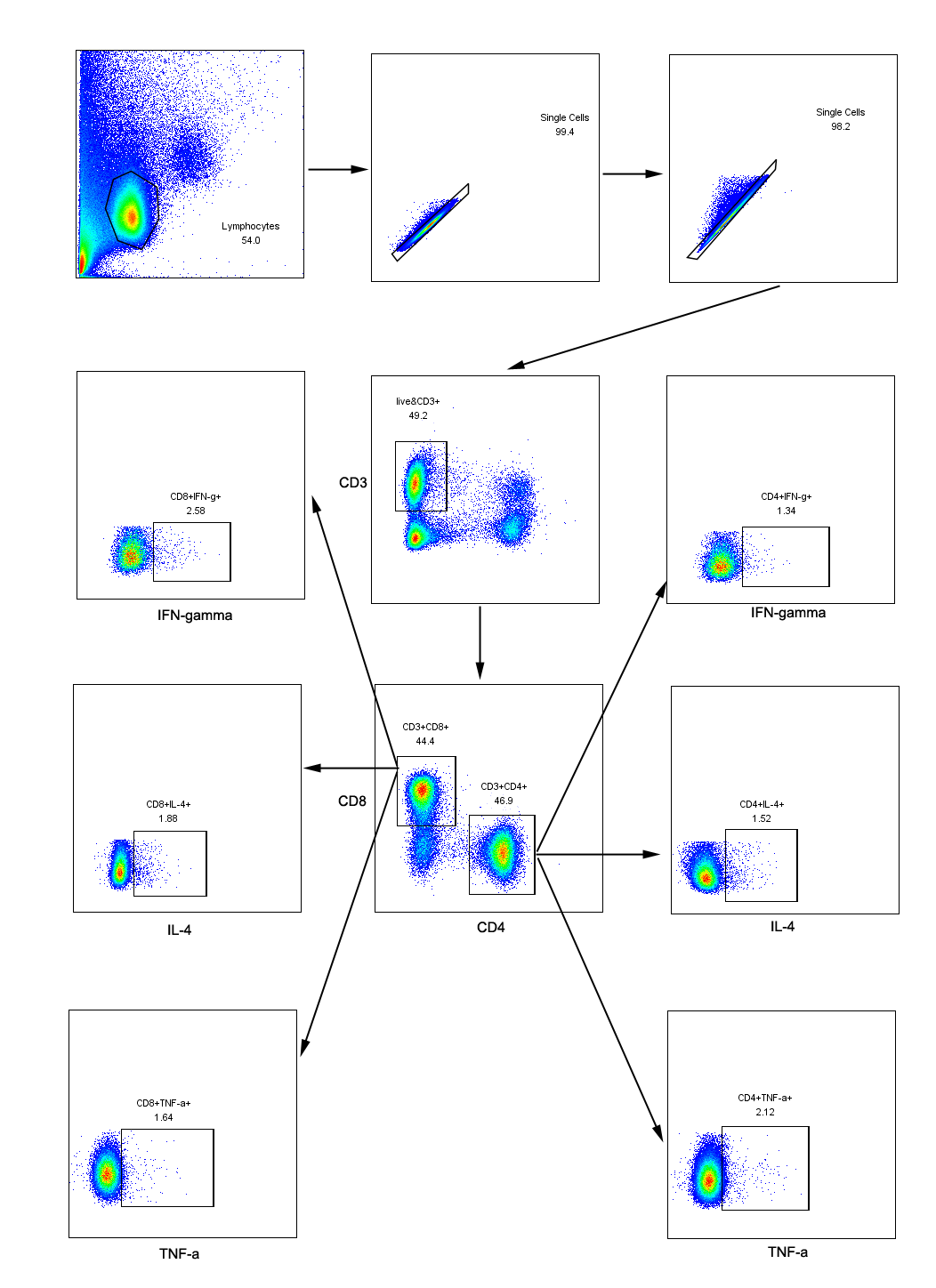
**

**Supplemental Figure 1. Gating strategy for flow cytometer analysis of production of intracellular cytokines.**

**
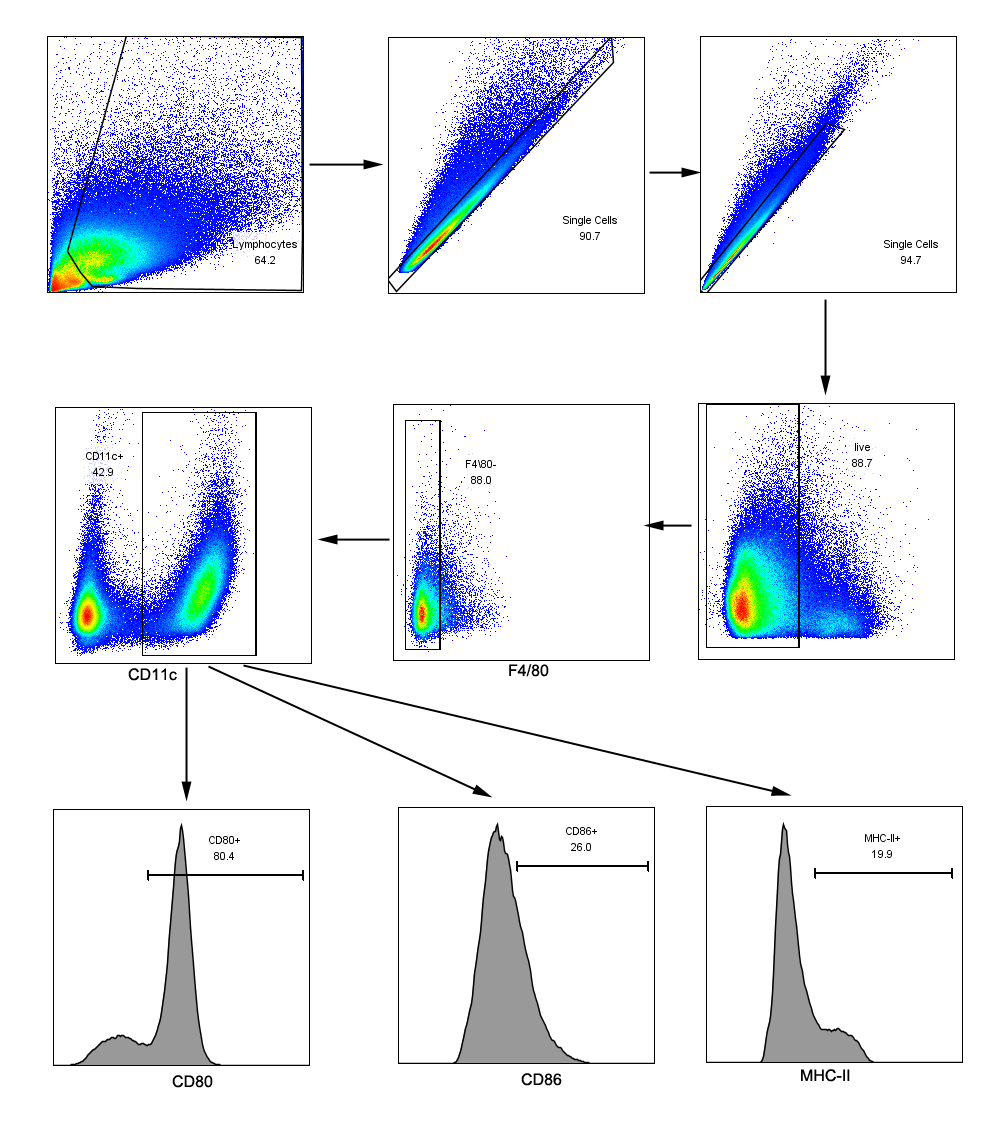
**

**Supplemental Figure 2. Gating strategy for flow cytometer analysis of lung dendritic cells.**

**
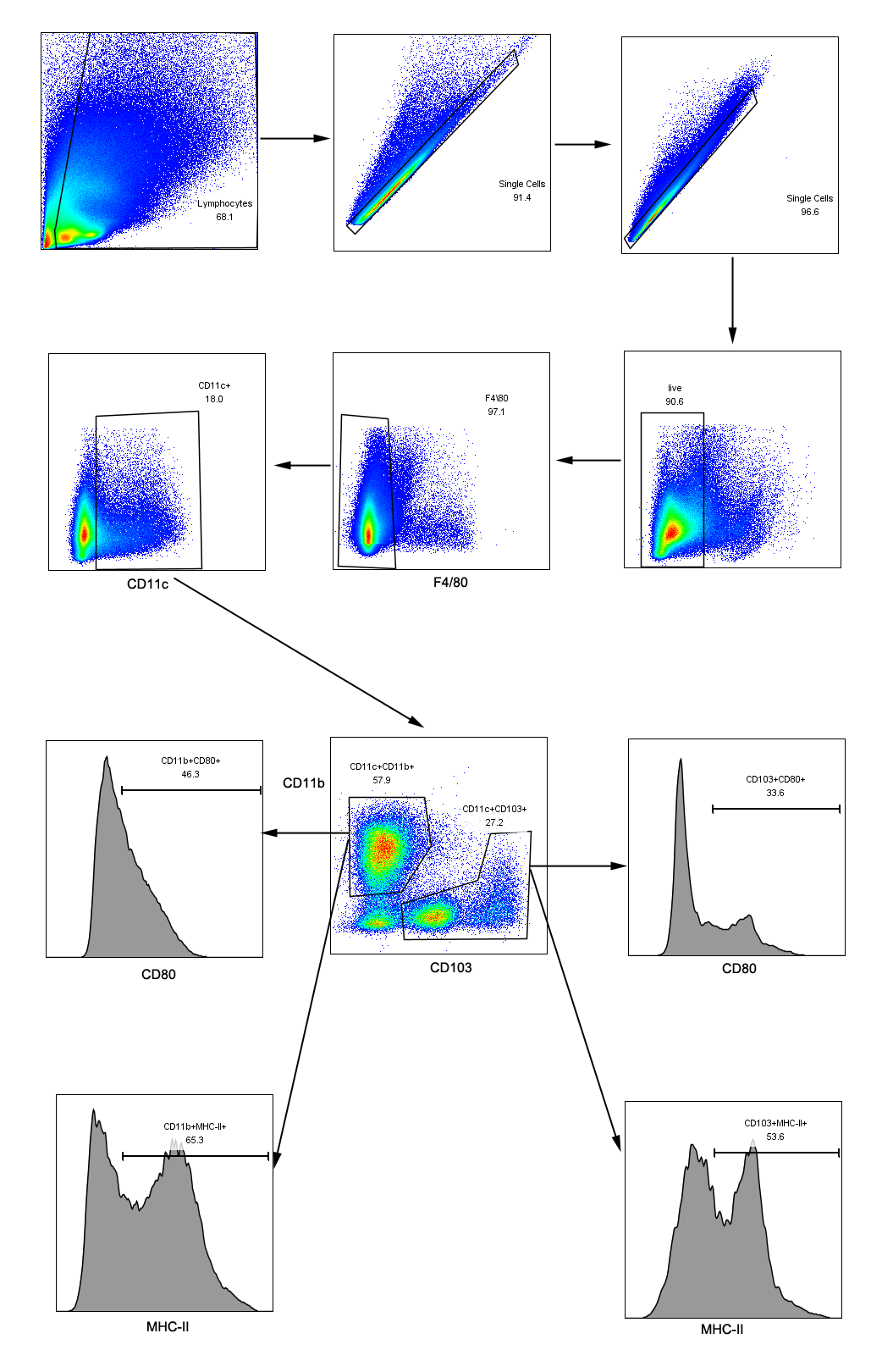
**

**Supplemental Figure 3. Gating strategy for flow cytometer analysis of CD11b^+^ and CD103^+^ lung dendritic cells.**

**
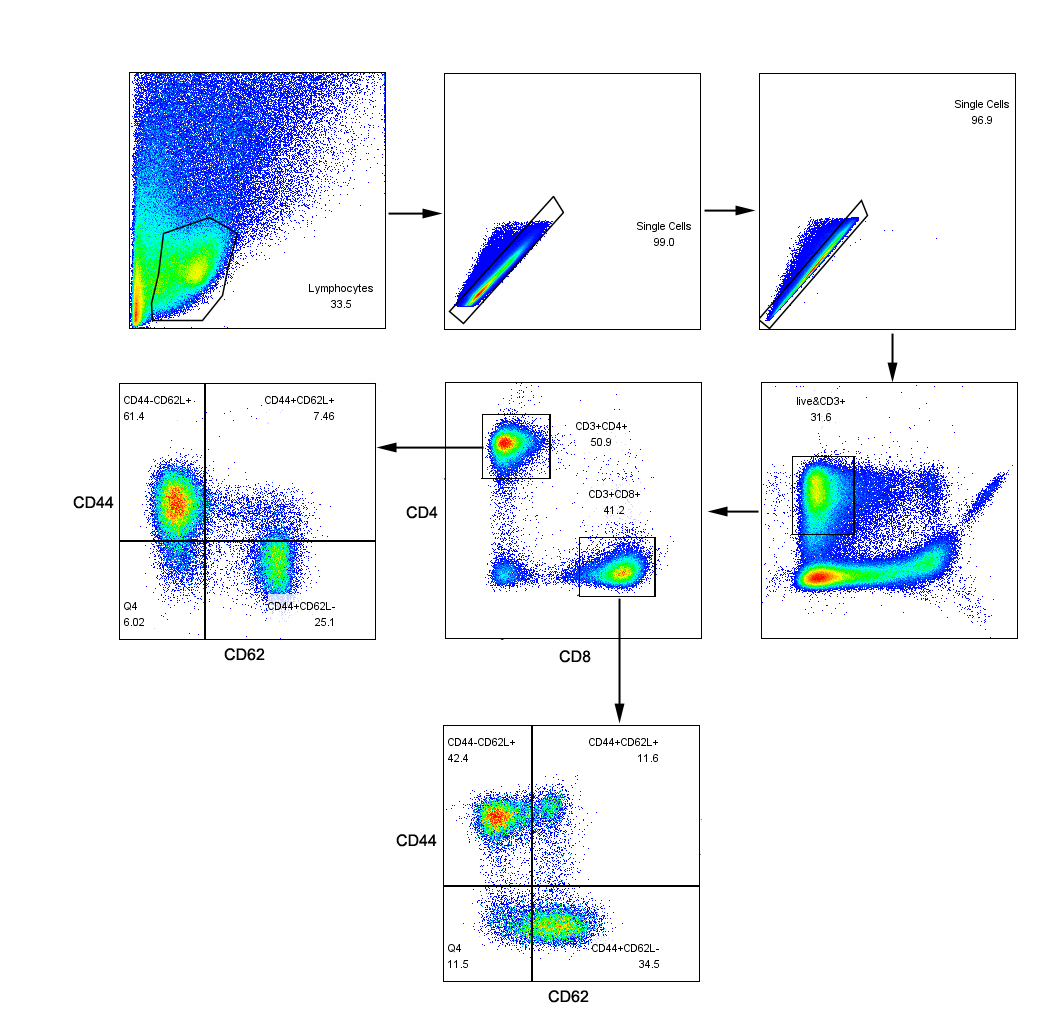
**

**Supplemental Figure 4. Gating strategy for flow cytometer analysis of effector memory T cells in lung.**

**
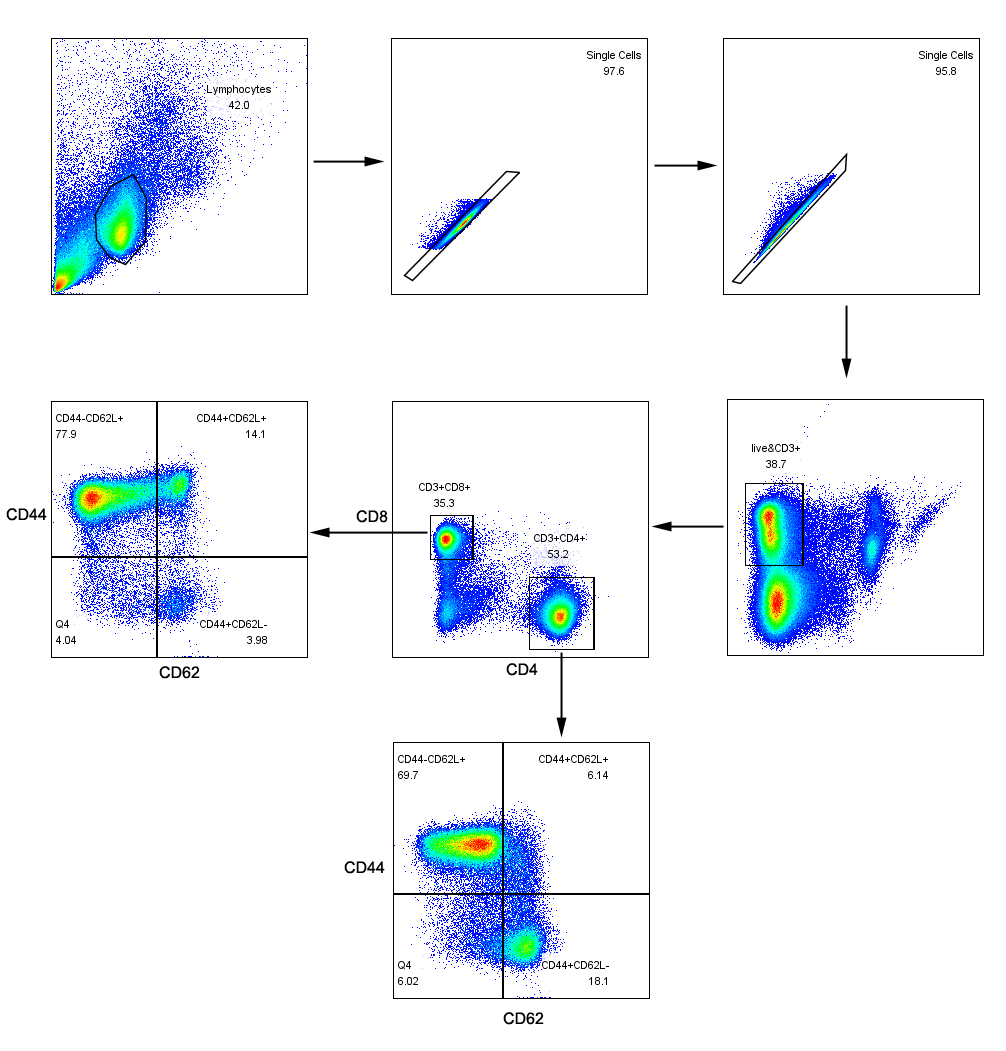
**

**Supplemental Figure 5. Gating strategy for flow cytometer analysis of effector memory T cells in spleen.**

**
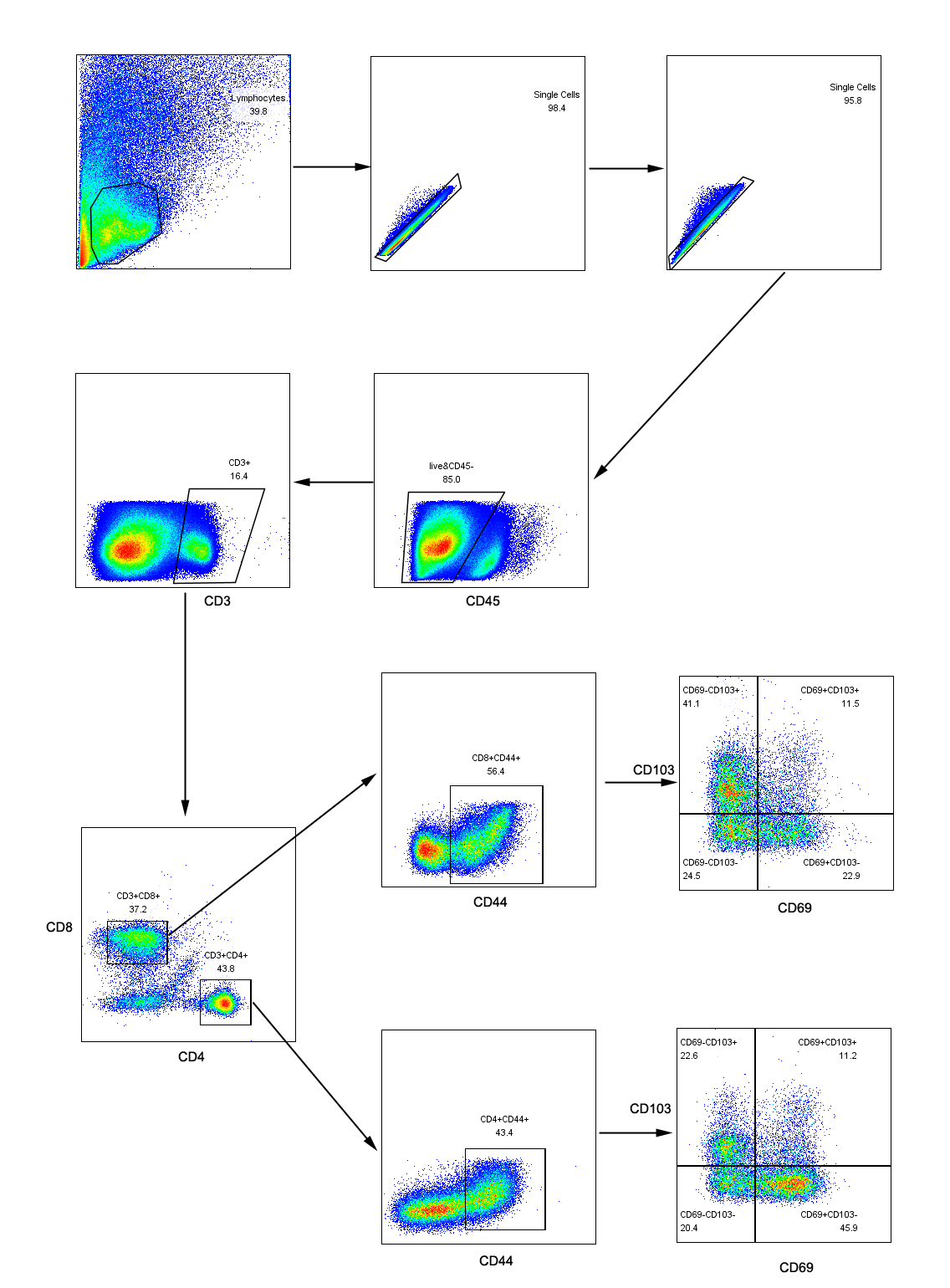
**

**Supplemental Figure 6. Gating strategy for flow cytometer analysis of lung resident memory T cells.**

**
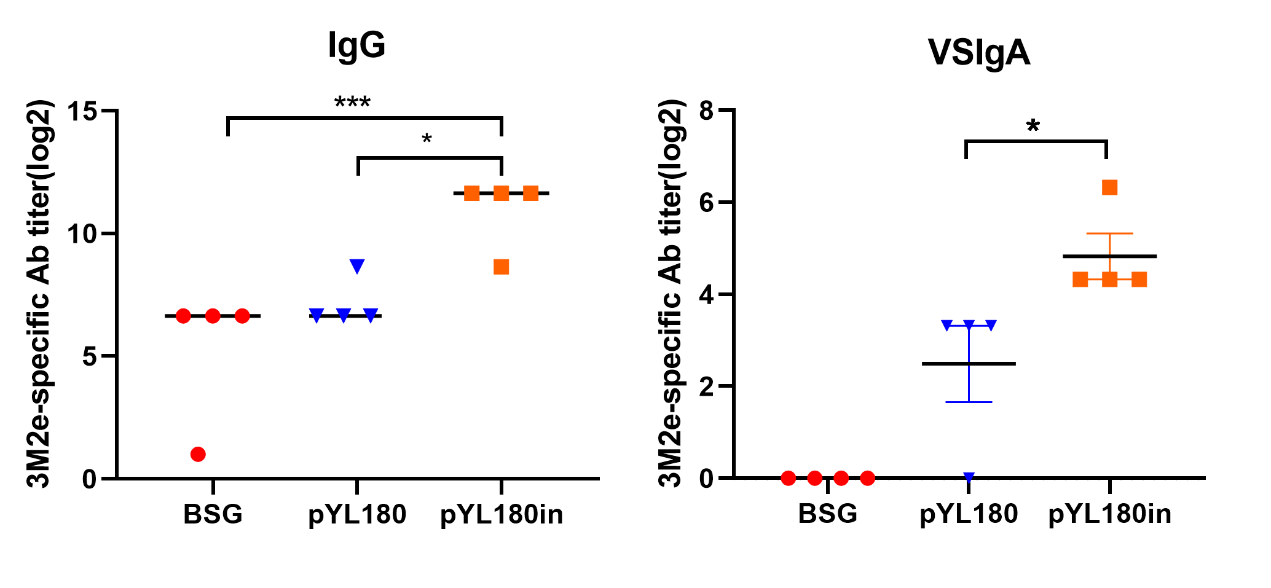
**

**Supplemental Figure 7. Serum IgG and IgA antibodies in vaginal washing samples from BALB/c mice.** BALB/c mice were orally administered *Salmonella* χ11802(pYL180) three times or two times plus an additional intranasal boost with purified 3M2e-ferritin nanoparticles (10 µg/20 µl). At 14 days post-3^rd^ immunization, serum and vaginal washing samples were collected, and the M2e-specific IgG and secretary IgA (VSIgA) antibodies were determined by ELISA (n=4, **P* < 0.05, ****P* < 0.001).
